# Enhanced artificial satellite search algorithm with memory and evolutionary operator for PID controller parameter estimation

**DOI:** 10.1038/s41598-025-24760-8

**Published:** 2025-11-12

**Authors:** Mohamed Issa

**Affiliations:** 1 Computer and Systems Department, Faculty of Engineering, Zagazig University, Zagazig, Egypt; 2 Faculty of Computer Science and Information Technology, Egypt-Japan University for Science and Technology, New Borg El Arab, Egypt

**Keywords:** PID controller, Control dynamic systems, Meta-heuristic optimization, Artificial satellite search algorithm, Evolutionary operators, Stochastic local search, Engineering, Mathematics and computing

## Abstract

The effective tuning of Proportional-Integral-Derivative (PID) controllers is crucial for industrial process control, but achieving optimal parameters for complex systems remains challenging. The recent Artificial Satellite Search Algorithm (ASSA) is strong in exploration but suffers from an imbalance between global and local search and a greedy selection strategy, leading to premature convergence. To overcome these limitations, this paper proposes an enhanced variant, MEASSA (Memory-based and Evolutionary-enhanced ASSA), which integrates a memory mechanism to preserve elite solutions, an evolutionary operator for guided population dynamics, and a stochastic local search for intensive refinement. Experimental evaluations on three dynamic systems are a DC motor, a three-tank liquid level system, and a fourth-order system which demonstrate MEASSA’s superior performance. The algorithm achieved the lowest Integral Absolute Error (IAE) values of 9.977, 9.0781, and 9.697, respectively, outperforming several benchmark metaheuristics. Time-domain and frequency-domain analyses further confirm its ability to minimize overshoot, improve settling time, and enhance system stability, validating MEASSA as a robust and accurate method for complex PID controller tuning.

## Introduction

PID controllers are widely employed in manufacturing industries for process control because they offer better effectiveness, robustness, and durability [1]. A PID controller regulates system stability, settling time, and response error through three parameters: proportional gain (${k}_{p}$), derivative gain (${k}_{d}$) and integral gain (${k}_{i}$). Proper tuning is critical in industrial settings, as it minimizes settling time, steady-state error, overshoot, and rise time for efficient performance. PID controllers are valued for maintaining setpoints despite disturbances, with broad applications from chemical reactors to motor speed control. Their simplicity makes them widely implementable and foundational for advanced control methods. The proportional term stabilizes the system, the integral term eliminates steady-state error, and the derivative term reduces error change, overshoot, and settling time [1].

The main limitation of PID controllers lies in optimally tuning the proportional (*k*_*p*_), integral (*k*_i_), and derivative (${k}_{d}$) gains, a complex and time-consuming task in nonlinear systems where poor tuning can cause instability. The tuning of PID controller parameters can be classified into three main categories: analytical methods, rule-based methods, and numerical methods [2]. The Ziegler–Nichols (ZN) method, the most common analytical approach for PID tuning, is widely used but often fails to deliver optimal performance [3].

Rule-based methods, which use heuristic or empirical rules, are often derived from experience or experimental data, such as fuzzy logic tuning and expert systems [4]. Rule-based and analytical PID tuning methods are limited by simplified models, fixed parameters, and reliance on heuristics or expertise, making them less accurate, adaptable, and consistent for real-world systems.

Numerical methods for PID tuning, such as meta-heuristics algorithms, which are computational optimization techniques to systematically search for the best controller parameters by minimizing a defined performance criterion [5]. Numerical methods overcome the limits of analytical and rule-based models by efficiently solving complex, nonlinear, and time-varying systems. Meta-heuristic algorithms enhance this by randomly exploring the search space to quickly find near-optimal solutions. Meta-heuristic algorithms imitate search strategies inspired by physics, human behavior, or nature. For example, the Sine–Cosine Optimization algorithm (SCA) [6] explores the search space by guiding agents toward the best-known regions using sine and cosine functions. Similarly, the Particle Swarm Optimization algorithm (PSO) [7] mimics the flocking behavior of birds in nature to optimize solutions. Metaheuristic algorithms have demonstrated significant effectiveness in addressing a wide range of complex engineering optimization problems across various domains, including bioinformatics [8–10], electric motor design [11], Solar energy systems [12–14], and passive suspension system optimization [15], among others [16]. Numerous metaheuristic techniques have been proposed in the literature for controller design.

**Fig. 1:**
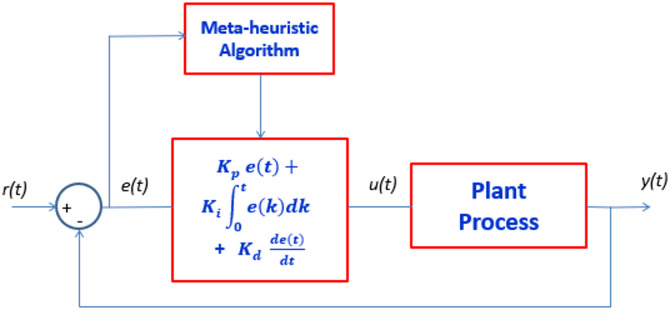
PID parameters tuning using a meta-heuristic algorithm [17]

As shown in Fig. 1, the meta-heuristic algorithm optimizes the parameters of the three PID controllers to minimize the rise time, overshoot, settling time, or the difference between the desired response (r(t)) and the actual response (y(t)). Eq (1) describes the integral of the absolute error (IAE), which represents the absolute difference between r(t) and y(t).

1 $$IAE= {\int }_{0}^{\infty }\left|y\left(t\right)-r(t)\right| dt$$

In the literature, various enhancements have been proposed for meta-heuristic approaches to optimize PID controller design. In [18], a PIDN controller was optimized with the Artificial Rabbit’s Optimization (ARO) algorithm for electric furnace temperature control, using adaptive tuning to improve accuracy and reduce overshoot. Comparative studies on DC motor control show that metaheuristic approaches, particularly Genetic Algorithms, outperform alternatives such as GWO, PSO, and ACO in improving rise time, settling time, and mean square error [19]. A study applied Dung Beetle Optimizer (DBO) and Ant-Lion Optimizer (ALO) to cascaded PID and FOPID controllers for Switched Reluctance Motor (SRM) speed control, achieving faster convergence and lower computational complexity than conventional methods [20]. In [21], PID-F controller optimized with the Spider Wasp Optimizer (SWO) was proposed for temperature control in continuous stirred tank reactors (CSTRs), addressing challenges of nonlinearity and time delays. In addition, several other meta-heuristic algorithms have been applied to optimize PID controller parameters to enhance the performance of DC motors, including Invasive Weed Optimization (IWO) [22], Flower Pollination Algorithm (FPA) [23], Firefly Algorithm [24], and Grey Wolf Optimization (GWO) [25]. For other applications, such as voltage regulator control, the Teaching Learning Based Optimization (TLBO) algorithm has been employed to optimize PID controller parameters [26]. Additionally, Differential Evolution (DE) and its enhanced variant, PSODE, have been used to optimize PID settings for three liquid level tank systems [27]. Also, Constrained Particle Swarm Optimization (CPSO) [28], dynamic Particle Swarm Optimization (dPSO) [29], opposition-based Henry Gas Solubility optimization algorithm (OBL-HGS) [30], and the improved Whale Optimization Algorithm (IWOA) [31]. According to the No-Free-Lunch (NFL) theorem [32] no single optimization algorithm can achieve optimal performance across all types of engineering problems. Consequently, enhanced variants of previously listed metaheuristic algorithms, developed to improve the performance PID controller.

The problem statement of this research study is optimizing the parameter estimation of the PID controller using the Artificial Satellite Search Algorithm (ASSA) [33] under the assumption of ideal and noise-free dynamic systems. ASSA is a new physics-based metaheuristic inspired by satellite dynamics. The ASSA models candidate solutions as satellites that adjust their positions to find optimal solutions by simulating both Medium Earth Orbit (MEO) and Low Earth Orbit (LEO) trajectories. This dual-orbit approach enhances the algorithm’s ability to explore and exploit the search space efficiently. It governs the equilibrium between gravitational and centrifugal forces crucial for stable satellite orbits. Within this framework, fundamental operators like gravitational force, mass, position, and velocity dictate satellite trajectories around Earth. Consequently, in the ASSA, each candidate solution (satellite) dynamically forms a unique relationship with Earth over time, thereby promoting more efficient exploration and exploitation of the search space.

ASSA achieves strong exploration capability through a well-integrated design combining several advanced mechanisms. It uses a logistic chaotic map for diverse initialization, adaptive parameters (β and γ) for dynamic, non-linear orbital fluctuations, and a time-decaying gravitational constant to shift smoothly from exploration to exploitation. Additionally, quantum-inspired qubits introduce probabilistic updates, and an orbit control mechanism alternates between global (MEO) and local (LEO) searches, ensuring broad coverage, flexibility, and escape from local optima, which makes ASSA highly effective for complex and high-dimensional optimization problems.

Despite its strengths, ASSA has two key limitations. First, its strong focus on exploration—driven by chaotic maps, adaptive parameters (β and γ), qubits, and orbit control—can reduce its ability to exploit promising regions, potentially slowing convergence in problems that require fine-tuning. Second, its greedy selection strategy, which always accepts better solutions, limits flexibility and increases the risk of premature convergence, as it cannot escape local optima like other algorithms that allow occasional acceptance of worse solutions.

To address the limitations of the original ASSA, an enhanced methodology is proposed by incorporating three key components: a memory mechanism, an evolutionary operator, and a stochastic local search, where this mechanism was applied successfully for enhancing the grey wolf optimizer [34]. The primary objective of these enhancements is to improve the balance between exploration and exploitation during the optimization process. The memory mechanism maintains a separate population that stores the best solutions found throughout the search, ensuring valuable solutions are not lost and enabling a more focused search around promising areas. Simultaneously, the evolutionary operator (based on Differential Evolution principles) guides the population with adaptive mutation and crossover, encouraging diversity in the early stages and fine-tuning solutions in later iterations through a dynamically controlled scaling factor.

In addition, a stochastic local search is employed to intensively refine high-quality solutions within the memory population. By adaptively generating trial solutions around the nearest neighbors of selected individuals, the local search further strengthens the algorithm’s exploitation capability while stabilizing convergence behavior. Together, these mechanisms effectively overcome the drawbacks of greedy selection and premature convergence, often observed in basic metaheuristic frameworks. The integration of these strategies results in a more robust and accurate optimization method, making the enhanced ASSA highly suitable for solving complex, multi-modal problems such as PID controller parameter tuning.

The key contributions of this work are outlined as follows:

1. ASSA optimized PID controller parameters for improved control performance.
2. MEASSA enhances ASSA with memory, evolutionary, and local search strategies.
3. MEASSA was benchmarked on three control systems against leading optimization algorithms.

The remainder of this paper is structured as follows: Sect. 2 provides the Artificial Satellite Search Algorithm. Section 3 describes the proposed enhanced version (MEASSA). Experimental results and a comprehensive discussion are presented in Sect. 4. Finally, the conclusions and key findings of the proposed work are summarized in Sect. 5.

## Artificial satellite search algorithm (ASSA)

**Fig. 2:**
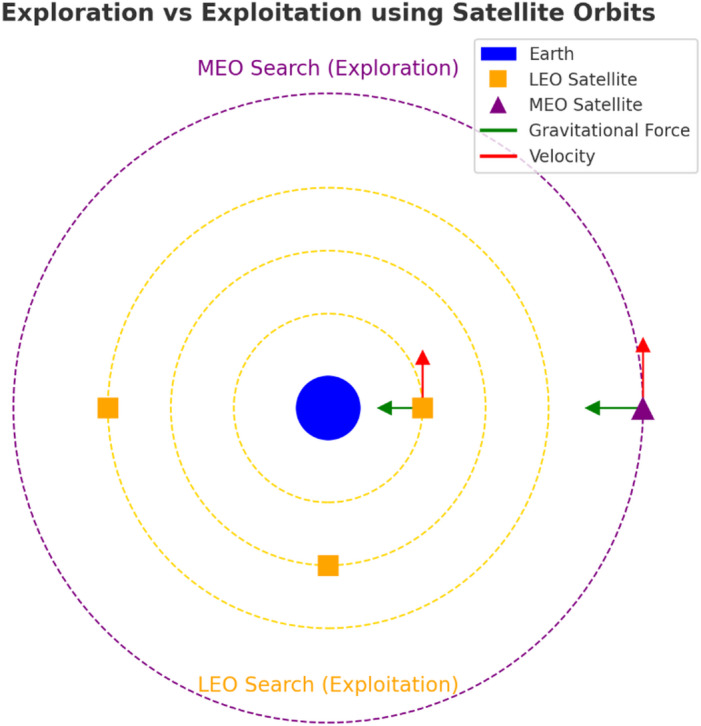
The simulation of satellite-like movements in the optimization domain [33]

The ASSA simulates fundamental physics principles by establishing hypothetical orbits for Earth and its satellites to represent the search space [35]. In this model, candidate solutions (satellites) experience varying conditions relative to Earth, which symbolizes the optimal solution, across different time instances. This dynamic interaction ultimately facilitates more efficient exploration and exploitation of the search space. The ASSA employs two primary satellite strategies for navigating the search space: MEO search, where satellites are positioned distantly from Earth to facilitate exploration, and LEO search, placing satellites closer to Earth for effective exploitation. Figure 2 illustrates how factors such as a satellite’s position and mass, its gravitational attraction to Earth, and its orbital velocity collectively influence the satellite’s trajectory relative to the optimal solution (represented by Earth).

In ASSA, each candidate solution, represented as a satellite, traverses an elliptical orbit with the “Earth” (representing the optimal solution) at one focus. Like other population-based meta-heuristics, ASSA begins with an initial set of satellite solutions whose fitness is evaluated. The algorithm then iterates, refining solutions based on an objective function, with the best solution in each iteration, like the current Earth. Significantly, the distance of each satellite from Earth is dynamically adjusted to reflect the passage of time within the optimization process.

### ASSA’s mathematical model

In the following, the mathematical modeling of ASSA.

#### Initialization process

Traditional random initialization of satellite populations often leads to slow convergence and a heightened risk of entrapment in local optima due to insufficient initial diversity. To overcome these drawbacks and reduce the likelihood of premature convergence, this study replaces the conventional random generation of satellites with a logistic chaotic map [36], as formulated in Eq. (2).

2 $$S_{i + 1} = \omega * S_{i} *\left( {1 - S_{i} } \right), 0 \le S_{i} \le 1$$

where $\omega$ represents a constant parameter and ${S}_{i}$ denotes the logistic chaotic position value corresponding to the i^th^ satellite and ${S}_{0} \varepsilon (0 , 1)$. Following the fitness evaluation of the initial population, ASSA designates the global best solution (${S}_{best}$), functionally represented as the Earth (${S}_{E}$).

#### Gravitational force

Eq (3) provides the calculation for the gravitational force between a satellite (${S}_{i}$) and the Earth (${S}_{best}$).

3 $${F}_{i(t)}= {G}_{(t)}*\left(\frac{{M}_{i}*{M}_{E}}{{\overline{R} }^{2}+\varepsilon }\right)*{r}_{(\text{0,1})}$$

G(t) is an exponentially decaying function, defined by Eq (4), which controls search precision over time (t). ${M}_{i}$ and ${M}_{E}$ denote the inertia masses of the satellite ${S}_{i}$ and Earth ${S}_{E}$, respectively, calculated using Eq (4) and Eq (6). $\overline{R }$ represents the Euclidean distance between ${S}_{i}$ and ${S}_{E}$, computed via Eq (7) and Eq (8).

4 $${G}_{(t)}={G}_{0}*{e}^{(-\alpha *\left(\frac{t}{T}\right))}$$

The constants ${G}_{0}$ and $\alpha$ are fixed, which are tuned via sensitivity analysis performed during the numerical experiments.

5 $${M}_{i(t)}= \frac{{fit}_{i(t)}-{worst}_{(t)}}{\sum_{i=1}^{NP}({fit}_{i\left(t\right)}-{worst}_{\left(t\right)})}$$

6 $${M}_{i(t)}=\left(\frac{{best}_{i\left(t\right)}-{worst}_{\left(t\right)}}{\sum_{i=1}^{NP}\left({fit}_{i\left(t\right)}-{worst}_{\left(t\right)}\right)}\right)*{r}_{(\text{0,1})}$$

Here, ${fit}_{i(t)}$ denotes the fitness value of satellite *i*. In the context of minimization problems, the ${worst}_{(t)}$ refers to the maximum fitness value, while ${best}_{i\left(t\right)}$ represents the minimum fitness value.

7 $${R}_{(t)}= \sqrt{\sum_{j=1}^{dim}{({S}_{best}-{S}_{i\left(t\right)})}^{2}}$$

8 $${\overline{R} }_{(t)}=\frac{{R}_{i(t)}-up({R}_{i\left(t\right)})}{up\left({R}_{i\left(t\right)}\right)-low({R}_{i\left(t\right)})}$$

Here, $dim$ represents the problem’s dimension, while $up({R}_{i\left(t\right)})$ and $low({R}_{i\left(t\right)})$ denote the upper and lower bounds of the Euclidean distance between satellite ${S}_{i}$ and Earth ${S}_{E}$, respectively.

#### Medium earth orbit (MEO) search

In the MEO phase, satellites are positioned at a considerable distance from Earth to enable efficient coverage of the entire search space. To enhance the exploration capabilities during this phase, the ASSA incorporates an adaptive factor (β) that simulates the natural variation in the satellite-to-Earth distance. This dynamic behavior evolves over time and is quantitatively defined by Eq (9).

9 $$\beta ={({e}^{{r}_{\left(\text{0,1}\right)}*\gamma })}^{-1}$$

where γ is a linearly decreasing control parameter ranging from 1 to −2, which is computed according to Eq (10)

10 $$\gamma =1+({r}_{\left(\text{0,1}\right)}*(\updelta -1))$$

where δ is a cyclic control parameter that gradually decreases from −1 to −2 over τ cycles throughout the entire optimization process, and is determined using Eq (11)

11 $$\delta =-1-(\frac{t\%\frac{T}{\tau }}{\tau })$$

where *t* denotes the current iteration number, *T* represents the maximum number of iterations, and *τ* indicates the total number of cycles within the entire optimization process.

**Fig. 3:**
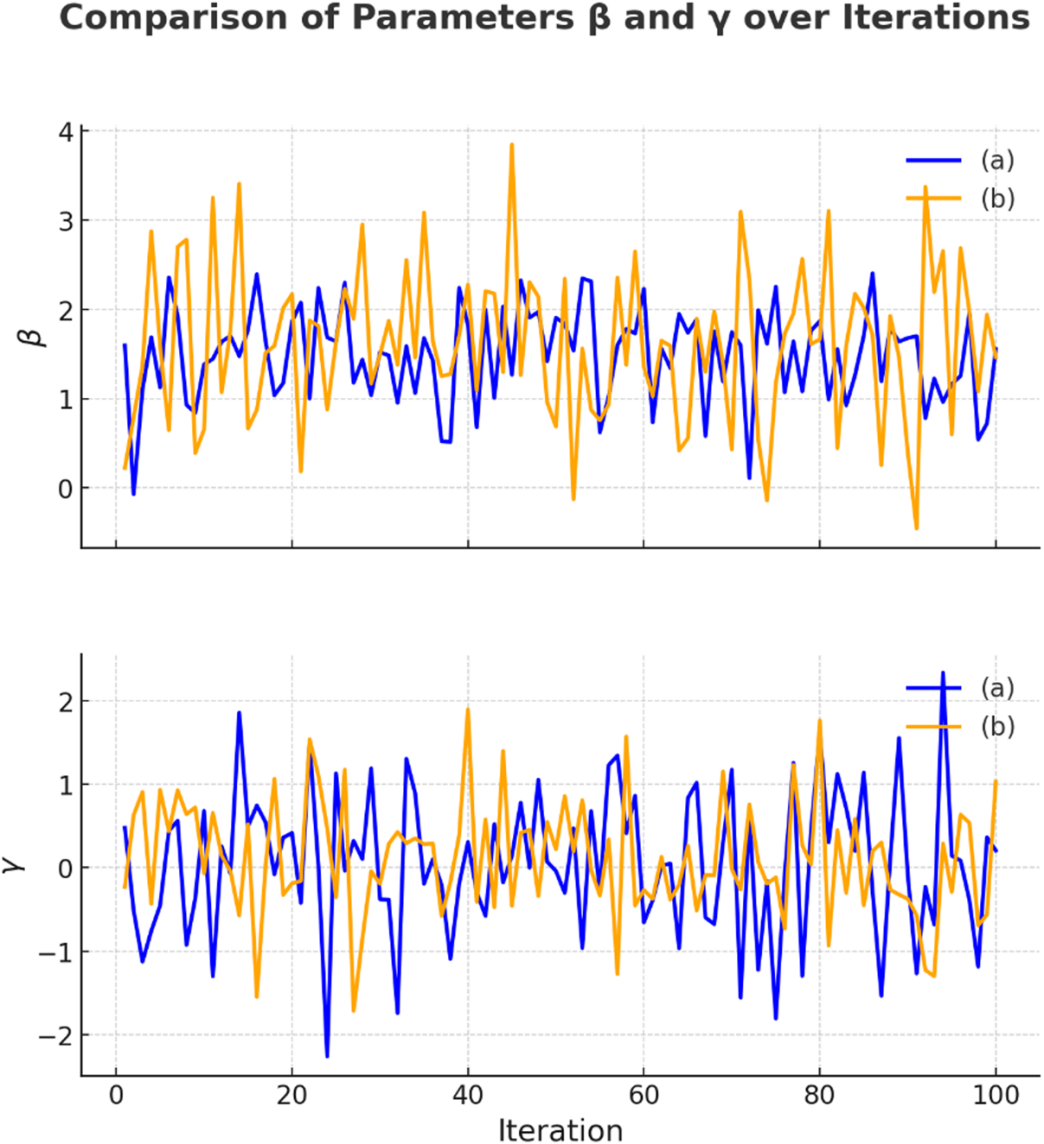
Simulation of the adaptive factors β and γ conducted over two independent runs [33]

Figure 3 illustrates the fluctuations of the adaptive factor across iterations. Higher values of the δ parameter correspond to broader exploration regions covered by a satellite, whereas lower δ values indicate a more focused search in the vicinity of the current best solution.

This principle is further enhanced by allowing it to fluctuate randomly, as defined by Eq (12) to improve the exploration capability of ASSA when calculating the satellite’s position relative to the Earth.

$${S}_{Mi(t+1)}={S}_{Mi(t)}*{q}_{\left[\text{0,1}\right]}+\left(1- {q}_{\left[\text{0,1}\right]}\right)*({S}_{mean\left(t\right)}+\beta *({S}_{mean\left(t\right)}-{S}_{Mi(t)}$$)) (12)

where ${S}_{mean\left(t\right)}$ represents the average of three solutions: ${S}_{i}$, ${S}_{best}$, and ${S}_{a}$; ${S}_{a}$ is a randomly selected solution from the population; *β* is an adaptive factor; and *q* ∈ [0, 1] denotes a qubit that transitions between states (0) and (1), contributing to the enhancement of the optimization process. According to the principles of quantum mechanics, a qubit can exist in a superposition of states 0 and 1 simultaneously. In the MEO search phase, the updated solution ${S}_{Mi(t+1)}$ is evaluated against the current solution, and the superior one is selected as the new global best solution ${S}_{best}$.

#### Low earth orbit (LEO) search

LEO artificial satellite search is influenced by MEO satellites, which have a broader coverage area. The velocity of a satellite depends on its location relative to the Earth. Kepler’s second law (the law of equal areas) and Kepler’s third law (the law of harmonies), outlined in Eq (13) and Eq (14)**,** govern the principles that determine the initial velocity and semimajor axis (*a*_*i*_) of the *i*^th^ satellite orbit at time *t*.

12 $${V}_{i(t)}=\sqrt{{G}_{0}*\left({M}_{i}+{M}_{E}\right)*\left|\frac{2}{R+\varepsilon }-\frac{1}{{a}_{i}+\varepsilon }\right|}$$

13 $${a}_{i(t)}=\sqrt[3]{\frac{{{T}_{i}}^{2}*{G}_{\left(t\right)}*({M}_{i}+{M}_{E})}{4{\pi }^{2}}}*{r}_{(\text{0,1})}$$

where ${T}_{i}$ denotes the orbital period of the i^th^ satellite, randomly generated according to a normal distribution; and ${r}_{(\text{0,1})}$ is a uniformly distributed random value in the range (0,1), introduced to enhance the diversity of the semimajor axis. The velocity of LEO satellites during their movement away from the Earth is determined by scaling the initial velocity by the distance between a randomly selected solution and the current solution, to gradually reduce the satellite’s velocity. However, a major limitation is the lack of diversity among solutions, which may restrict the satellites’ ability to escape local optima over time, as the current solution continues to evolve. To address this limitation, ASSA incorporates a step size derived from the range between the lower and upper bounds of the optimization problem.

Additionally, when satellites approach the Earth, their velocity is computed by multiplying the initial velocity by the distance between the current solution and a randomly selected solution. This mechanism enhances the diversification of ASSA’s search strategies. As a result, although this approach promotes exploration, it may also lead to reduced population diversity over time, potentially causing a decrease in velocity during the optimization process. To preserve satellite velocity throughout the optimization process and prevent stagnation in local minima, an additional step is incorporated based on the distance between the lower and upper bounds of the search space. This enhancement is implemented using Eq. (15) and Eq (16).

14 $${v}_{i(t)}={r}_{(\text{0,1})}*{V}_{i\left(t\right)}*\left({S}_{a\left(t\right)}-{S}_{Li\left(t\right)}\right)+{r}_{\left(\text{0,1}\right)}*q\_dir*(1-\overline{{R }_{\left(t\right)})}*({U}_{B}-{L}_{B})$$

15 $${v}_{i(t)}={r}_{(\text{0,1})}*{V}_{i\left(t\right)}*\left({S}_{Li\left(t\right)}-{S}_{b\left(t\right)}\right)+{(1-r}_{\left(\text{0,1}\right)})*{V}_{i\left(t\right)}*\left({S}_{a\left(t\right)}-{S}_{b\left(t\right)}\right)+ {r}_{\left(\text{0,1}\right)}*q*(1-\overline{{R }_{\left(t\right)})}*({U}_{B}-{L}_{B})$$

where ${S}_{b}$ and ${S}_{a}$ denote two solutions randomly selected from the population; ${U}_{B}$ and ${L}_{B}$ represent the upper and lower bounds of the search space, respectively; and $q\_dir$ is the qubit direction operator employed in Eq (17) to enhance the orbital movement of satellites. This operator is utilized to alter the search direction, thereby increasing the likelihood that satellites effectively scan the search space. Consistent with the principles of quantum mechanics, the qubit can exist in a superposition of both 0 and 1 states simultaneously, enabling a more flexible and probabilistic exploration behavior.

16 $$q_{dir} = q_{{\left[ {0,1} \right]}} *\to _{dir}$$

where ${q}_{\left[\text{0,1}\right]}$ represents a qubit capable of transitioning between the quantum states (0) and (1), to enhance the optimization process by introducing probabilistic behavior and promoting exploration. This process involves comparing two random values: if the first ${r}_{(\text{0,1})}$ is greater than the second, the qubit is assigned a value of 1; otherwise, it is set to 0. The symbol $\underset{dir}{\to }$ represents the rotational direction of a satellite around the Earth, which can be either counterclockwise or clockwise. If ${r}_{(\text{0,1})}$ < 0.5, $\underset{dir}{\to }$ is set to 1 (indicating counterclockwise rotation); otherwise, it is set to −1 (indicating clockwise rotation).

Assigning a new position to a satellite involves an additional step size, calculated as the product of the distance between the current satellite and the Earth and the gravitational force. This adjustment enables ASSA to effectively exploit the regions surrounding the current best solution, leading to improved performance with fewer function evaluations. Typically, satellite velocity serves as the primary search operator in ASSA when a satellite is moving away from the Earth. However, this velocity is influenced by the Earth’s gravitational force, which aids in the fine-tuned exploitation of areas near the optimal solution. As a satellite approaches Earth, its velocity increases substantially, enabling it to counteract the intensifying gravitational pull. In this scenario, velocity acts as a mechanism to escape local optima, particularly when the best-so-far solution (i.e., the Earth) corresponds to a local minimum. Therefore, the gravitational attraction of the Earth functions as an exploitation operator, guiding ASSA to challenge the current best solution in pursuit of potentially superior alternatives. This behavior is mathematically described in Eq (18).

17 $${S}_{Li\left(t+1\right)}={S}_{Li\left(t\right)}+\underset{dir}{\to }* {v}_{\left(t\right)}+\left(1-{q}_{\left[\text{0,1}\right]}\right)*{F}_{i\left(t\right)}* \left({S}_{a\left(t\right)}-{S}_{Li\left(t\right)}\right)$$

#### Orbit control mechanism

**Fig. 4:**
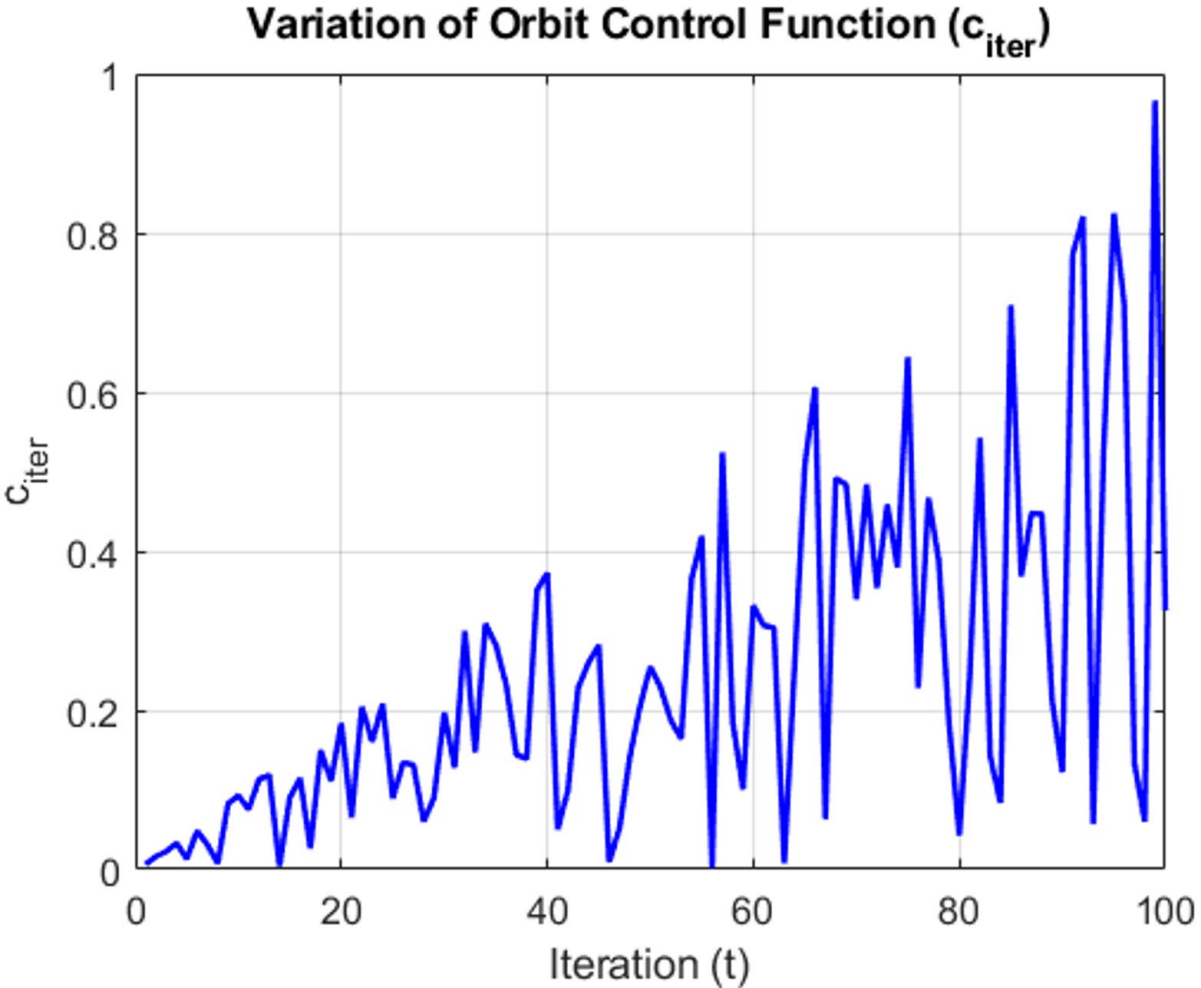
Illustration of the orbit control computation [33]

Satellites operate concurrently within the same orbit and across different orbits to collaboratively search for the desired target solution. Initially, MEO satellites determine their positions within the MEO orbit, while LEO satellites actively search for improved solutions. To simulate this coordinated behavior, an orbit control mechanism, denoted as ${c}_{iter}$, is introduced. This variable varies randomly between 0 and 1 over successive iterations, ensuring that each search cycle is executed efficiently. The orbit control function is mathematically defined in Eq (19), and the variation in the orbit control function across progressive iterations is illustrated graphically in Fig. 4.

18 $${c}_{iter}=\left|\left(2*{r}_{\left(\text{0,1}\right)}-1\right)*(\frac{t}{T})\right|$$

where *t* denotes the current iteration number *i*, and *T* represents the total number of iterations in the optimization process.

The main advantages of ASSA, which motivate us to use it, are as follows:

1. Diversity of Generated Solutions Using Logistic Chaotic Map

The initialization phase is crucial in metaheuristic algorithms. ASSA enhances it by using a logistic chaotic map Eq (2) instead of random initialization, introducing structured randomness that ensures diverse satellite positions. This boosts early exploration and reduces the risk of premature convergence and local optima entrapment.

2. Dynamic Adjustment of the Gravitational Constant over time

Eq. (3) in ASSA dynamically reduces the gravitational constant over time, controlling the attraction between satellites and the global best solution. This exponential decay enables a strategic shift from exploration in early iterations to exploitation later, enhancing convergence by balancing global and local search.

3. Adaptive parameters β and γ

In the MEO search phase, ASSA uses adaptive parameters β and γ (from Eq (9) and Eq (10) to simulate orbital fluctuations. These parameters control how far a satellite deviates from the average of itself, a random peer, and the best solution. This cyclic, non-linear adaptation enhances exploration diversity and dynamically adjusts the search intensity based on progress, preventing stagnation.

4. Incorporation of quantum-inspired principles

A key feature of ASSA is its use of quantum-inspired qubits (Eq. 12), which can exist in superposition, enabling probabilistic position updates. The qubit value q [0,1] controls the influence of current, peer, and best solutions. This stochastic behavior enhances exploration and helps the algorithm escape local optima by encouraging diverse and flexible search paths.

5. Strength Exploration

ASSA’s strong exploration ability stems from its integrated design: a chaotic map for diverse initialization, time-varying gravitational control, adaptive parameters (β and γ), and an orbit control mechanism that switches between global (MEO) and local (LEO) search. Combined with quantum-inspired qubits, these components ensure high diversity, effective local escape, and broad search coverage—ideal for complex, high-dimensional problems.

However, the main drawbacks of ASSA are as follows:

1. High Diversity May Limit Exploitation

ASSA’s emphasis on exploration (through chaotic maps, adaptive parameters (β and γ), qubits, and orbit control) can limit its ability to exploit. While these mechanisms help escape local optima, they may hinder convergence in problems requiring fine-tuning. Excessive variation may lead the algorithm to continue exploring when focused exploitation would yield faster and more precise results.

2. Greedy Selection Strategy

Another limitation of ASSA is its greedy selection strategy, where it always accepts better solutions without allowing worse ones. While the greedy strategy in metaheuristics helps accelerate convergence by always accepting better solutions, it often leads to significant drawbacks such as premature convergence, reduced population diversity, and limited exploration of the search space. By focusing solely on immediate improvement, the algorithm may get trapped in local optima, especially in complex or multi-modal landscapes, and lacks the flexibility to explore suboptimal regions that could lead to better solutions later.

## The proposed MEASSA

To overcome the main drawbacks of ASSA, a memory mechanism for preserving better historical solutions and an evolutionary operator is embedded, as described in the following subsections.

### Evolutionary operators

The evolutionary operator is inspired by the mutation and crossover mechanisms of DE, and it is applied to the explorer swarm (the main working population). Its main idea is to evolve individuals by combining information with the best solution and other members of the population. The evolutionary operator implements the DE/best/1/bin strategy.

The mutation operator is applied according to Eq (20)

19 $${{V}_{j}}^{(t+1)}={S}_{j}+ F ({S}_{best}-{S}_{j})$$

Where ${S}_{best}$ and ${S}_{j}$ represents the best solution and a randomly selected solution, and $F$ as represented in Eq (21) is a dynamic scaling factor that is developed to enhance the balance between exploration and exploitation phases.

20 $$F={F}_{min}+\left({F}_{max}-{F}_{min}\right)\frac{T-t}{T}$$

where ${F}_{max}$, ${F}_{min}$ represent the maximum and minimum values of $F$ and $T$ and $t$ represent the number of total iterations and current iterations iteratively.

The crossover operator is performed by mixing ${V}_{j}$ and ${S}_{j}$ as shown in Eq (22)

21 $${{U}_{j}}^{(t+1)}= \left\{\begin{array}{c}{{V}_{j}}^{(t+1)} if rand\le Pc \\ {{S}_{j}}^{\left(t\right)} Otherwise\end{array}\right.$$

where U_j_ is a test individual parameter and P_c_ is a crossover probability control.

The selection is performed according to Eq (23)

22 $${{S}_{j}}^{(t+1)}= \left\{\begin{array}{c}{{U}_{j}}^{(t+1)} if func({{U}_{j}}^{\left(t+1\right)} )\le func({{S}_{j}}^{\left(t\right)} ) \\ {{S}_{j}}^{\left(t\right)} Otherwise\end{array}\right.$$

The main benefit of embedding the evolutionary operator improves exploration in the early stages through a high scaling factor, enabling the algorithm to escape local optima, while gradually shifting toward exploitation in later stages by narrowing the search around the best solutions. This operator maintains population diversity, prevents premature convergence, and ensures only better offspring are retained through greedy selection. By generating competitive solutions and adapting the search strategy over time, the evolutionary operator plays a critical role in balancing exploration and exploitation, resulting in faster convergence and improved solution accuracy.

### Memory mechanism

No personal best or memory for saving the best wolves found so far during the iterations. The current population represents the explorer, while the memory of this population with equal size to store the better solution found during the iterations. After each iteration, the memory population is updated with the better value from the corresponding explorer population.

The main benefit of this mechanism is enhancing the exploitation behavior of the algorithm by preventing the loss of good solutions and promoting intensive search around promising areas and avoiding the drawbacks of greedy strategy mechanisms.

### Local search

The stochastic local search significantly enhances exploitation by intensively refining the neighborhood around high-quality solutions, specifically the top 50% of solutions in the memory swarm. By generating trial solutions based on the position of a solution and its nearest neighbor, local search enables directionally adaptive local exploration, helping the algorithm to fine-tune solutions with greater accuracy.

This targeted search avoids redundant exploration of low-quality areas, making the search process more efficient and focused. Additionally, local stabilizes convergence in later iterations by reducing unstable behavior and complements the global exploration introduced by the evolutionary operator and memory mechanism, ultimately improving the algorithm’s ability to locate and converge on the global optimum.

The stochastic local search is performed by finding the nearest solution (S_n_) to the current solution (S_i_) in the memory population based on the Euclidean distance. Then a temporary solution is generated according to Eq (24):

23 $${{S}_{TP}}^{Mem}={{S}_{i}}^{Mem}+ rand \left(\text{0,1}\right)* ({{S}_{i}}^{Mem}-{{S}_{n}}^{Mem})$$

If the cost function of the generated temporary solution is better than that of the current solution in the memory population, it replaces the current one; otherwise, it is discarded.

**Algorithm 1:**
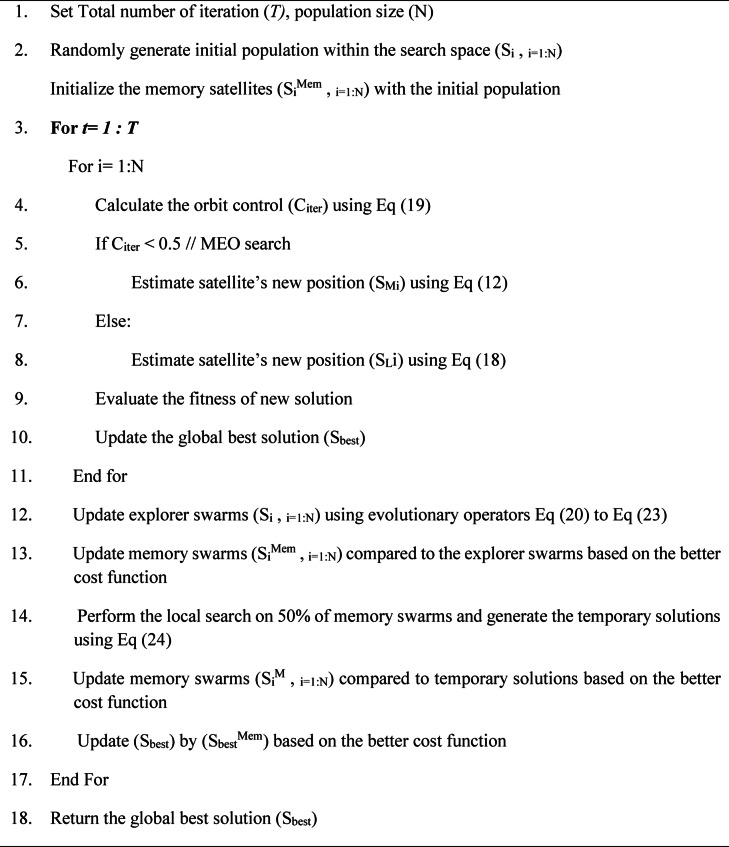
MEASSA

The computational complexity analysis reveals fundamental differences between the original ASSA and its enhanced version MEASSA.

For both algorithms, the key parameters governing time complexity are N (population size), T (maximum number of iterations), and D (problem dimension, fixed at D=3 for PID controller parameter optimization). The original ASSA exhibits linear complexity of O (T × N × D), which simplifies O (T × N) given the constant dimensionality of the PID tuning problem. This efficiency stems from ASSA’s core operations, including gravitational force calculations, orbital position updates, and fitness evaluations, all scaling linearly with population size.

In contrast, MEASSA introduces three significant enhancements: evolutionary operators, memory mechanisms, and stochastic local search, which substantially alter its computational profile. The evolutionary operator contributes O (T × N × D) complexity through mutation and crossover operations, while the memory mechanism adds minimal overhead of O (T × N). However, the stochastic local search proves computationally intensive, requiring nearest-neighbor calculations for the top 50% of solutions that yield O (T × N^2^ × D) complexity.

Consequently, MEASSA’s overall time complexity becomes O (T × N^2^ × D), or simplified to O (T × N^2^), representing a quadratic relationship with population size. This complexity of trade-off reflects the fundamental balance in metaheuristic optimization, where MEASSA sacrifices computational efficiency for enhanced solution quality through more intensive exploitation mechanisms, making it particularly suitable for applications where solution accuracy outweighs computational cost considerations.

## Experimental results and discussion

**Fig. 5:**
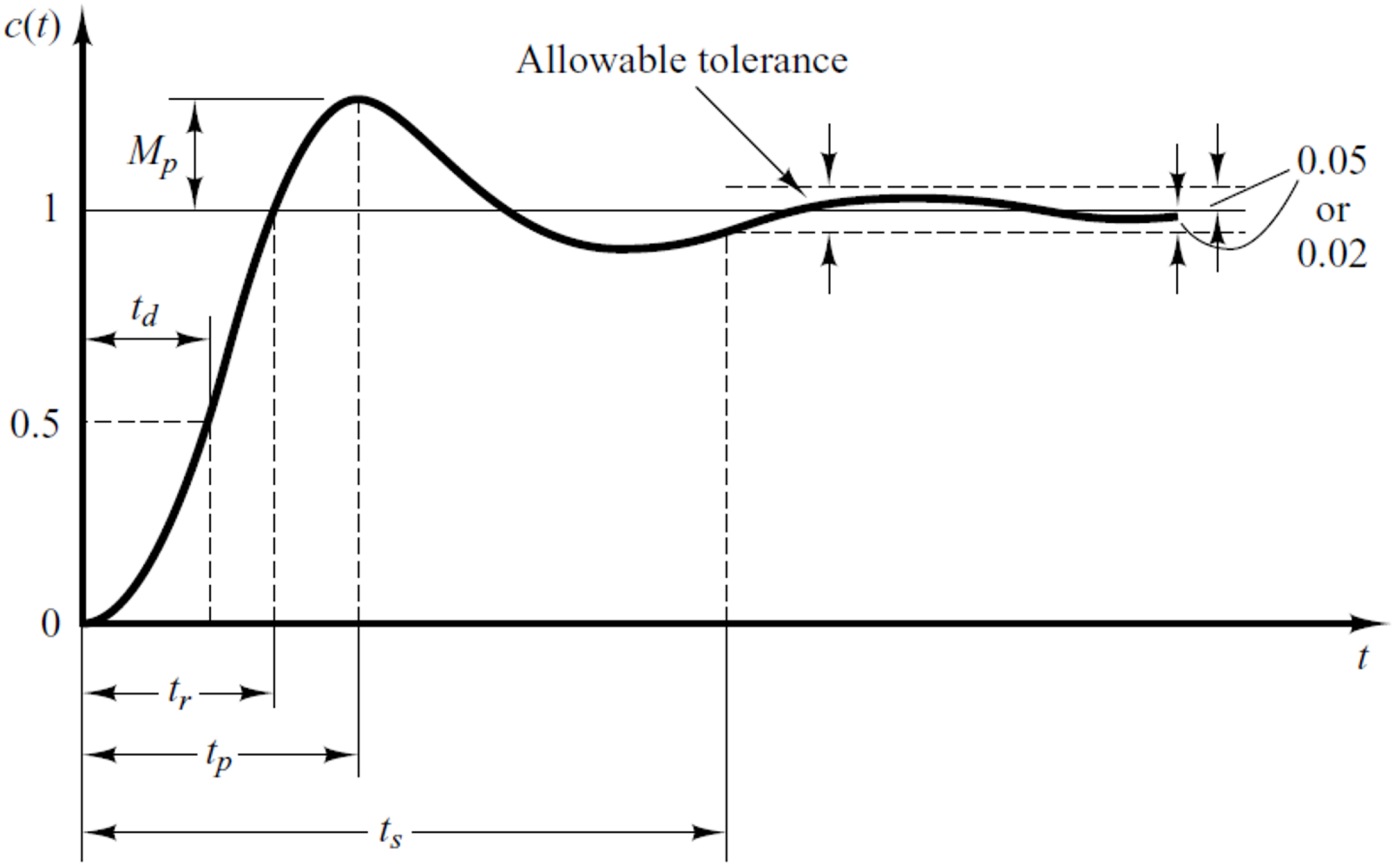
Time domain specification of controlled process response

The step response characteristics of a controlled process in the time domain include delay time, rise time, peak time, settling time, and overshoot, as illustrated in Fig. 5. These characteristics are defined as follows [1]:

1. Delay Time (t_d_): The time it takes for the response to initially reach 50% of its final value.
2. Peak Time (t_p_): The duration required for the response to attain its first peak value due to overshoot.
3. Rise Time (t_r_): The time needed for the response to increase from 10% to 90% of its final value.
4. Settling Time (t_s_): The time it takes for the response to remain within a specific percentage (typically 2%) of the final value.
5. Overshoot (M_p_): The highest value reached by the response curve above the final value, usually expressed as a percentage over unity.

Experimental tests were conducted on three systems. The first involved DC motor speed control, a setup frequently used in numerous related studies [11, 22, 25, 30, 31, 37]. The second system focused on regulating the liquid level in a series of three interconnected tanks [27]. The third system is the more complex one, which is a fourth-order transfer function. All simulations were performed under ideal, noise-free conditions to provide a clear baseline for comparing the optimization algorithms. The PID controller was implemented in its standard form without a derivative filter. The fitness function was used to evaluate solutions based on IAE using Eq (1), which represents the main objective. By targeting IAE, the controller indirectly pushes the system toward shorter rise and settling times, reduced overshoot, and quicker error recovery, although it does not guarantee optimal values of each metric individually. The experimental results were compared with relevant studies, including SCA [6], GWO [25], PSO [38], IWO [22], mJS [39], PSO-ACO [40], OBL-HGS [1] and CMA-ES [41]. The MEASSA algorithm was configured with a population size of 30 (as determined by sensitivity analysis) and a maximum of 50 iterations, corresponding to a stopping criterion of 1500 function evaluations. The evolutionary operator used a dynamically decaying scaling factor (Fmax=0.9, Fmin=0.2) via Eq. 21 and a crossover probability of Pc=0.9 determined via sensitivity analysis. All statistical results are based on 30 independent runs per tested system.

### DC motor speed regulator system [25]:

The transfer function of the DC motor closed-loop speed control system with sampling time (T_s_ = 10ms) is given as shown in Eq (24), while the state space representation is shown in Eq (25). The parameter values of the DC motor used in the case study are presented in Table 1 [25].

**Table 1:** Parameters of DC motor [25]

Parameter	Value
R_a_	0.4 Ω
L_a_	2.7 mH
J	0.0004 kg. m^2^
D	0.0022 N.m.sec/rad
K	15 e−03 kg. m^2^/s^2^ A
K_b_	0.05 V.s

R_a_ denotes the armature resistance, L_a_ is the inductance of the armature winding, J represents the equivalent moment of inertia of the motor and load referred to the motor shaft, D stands for the equivalent friction coefficient of the motor and load referred to the motor shaft, K is the motor torque constant, and K_b_ refers to the back EMF constant.

24 $${G}_{1}\left(S\right)= \frac{15}{{1.08 s}^{2}+6.1 s+1.63}$$

25 $$\left\{\begin{array}{c}{\dot{x}}_{1}\left(t\right)={x}_{2}\left(t\right)\\ {\dot{x}}_{2}\left(t\right)=-1.51 {x}_{1}\left(t\right)-5.65 {x}_{2}\left(t\right)+ u\left(t\right)\\ y\left(t\right)=13.89 {x}_{1}(t)\end{array}\right\}$$

Table 2 shows the optimal PID controller parameter values for DC motor speed regulation achieved using the MEASSA algorithm, compared against standard ASSA and other related algorithms included in the comparative study. The MEASSA algorithm focuses on optimizing a single objective (IAE) by identifying the PID parameters that minimize this value. Additional performance metrics such as settling time, rise time, and overshoot were also evaluated based on the PID parameters estimated by MEASSA and the other algorithms.

**Table 2:** Step Response Metrics and Best IAE for Heuristic Algorithms for DC Motor Speed Regulator.

Method	Kp	Ki	Kd	Set Time (Sec)	Rise Time (Sec)	Over-shoot %	IAE
SCA [42]	4.938	0.166	0.17	1.0819	0.1842	16.806	16.312
GWO [25]	11.283	0.178	0.326	0.6588	0.107	25.762	14.903
PSO [7]	17.895	8.389	1.561	0.3353	0.0688	8.1559	15.519
IWO [22]	8.628	3.856	0.17	1.0172	0.1224	32.804	15.14
mJS [39]	15.316	7.578	2.024	0.2869	0.0677	2.8625	11.285
PSO-ACO [40]	18.924	5.037	17.577	0.0252	0.0096	0	12.629
OBL-HG [30]	16.602	1.393	1.04	0.3248	0.079	12.948	14.086
CMA-ES [41]	14.202	15.261	1.728	0.5311	0.0739	5.2494	13.306
ASSA	19.583	16.951	5.115	0.0886	0.0335	0.2226	14.501
MEASSA	17.151	0.347	2.11	0.2211	0.0636	2.6982	**9.977**

The significance of bold value in Table 2 is just an indication of minimum value on the column.

Compared to ASSA and other popular metaheuristic algorithms (e.g., PSO, IWO, CMA-ES, OBL-HGS), MEASSA demonstrated superior performance across all key performance indicators. Specifically, MEASSA achieved the lowest IAE value (9.977) among all tested algorithms, confirming its improved convergence behavior and superior ability to minimize steady-state and transient errors. In contrast, the original ASSA algorithm yielded a higher IAE of 14.501, reflecting its strong exploration capabilities but limited exploitation due to a lack of memory and local refinement.

The improved results of MEASSA are attributed to its balanced exploration–exploitation strategy, achieved through a combination of mechanisms that enhance both global and local search capabilities. The evolutionary operator introduces diversity and prevents the population from getting trapped around local optima, while the memory mechanism maintains a parallel swarm of the best-found solutions, enabling intensified search around promising regions.

Additionally, the stochastic local search refines top-performing solutions for more accurate convergence. Together, these enhancements effectively overcome the limitations of ASSA, such as its greedy selection strategy and overemphasis on exploration. MEASSA introduces adaptive convergence behavior, promoting exploration in early iterations and shifting toward exploitation in later stages through a dynamic scaling factor and targeted local refinement.

**Fig. 6:**
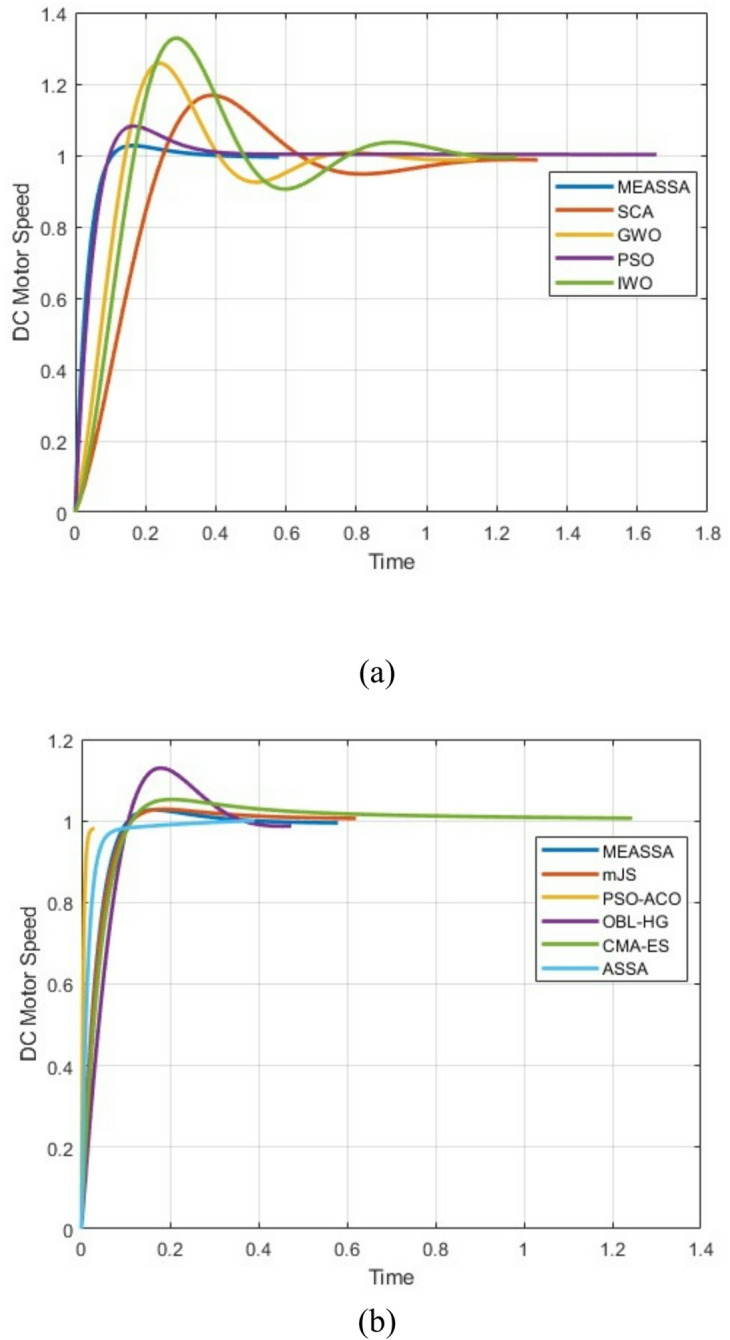
DC Motor Speed Response Over Time (in Seconds)

**Fig. 7:**
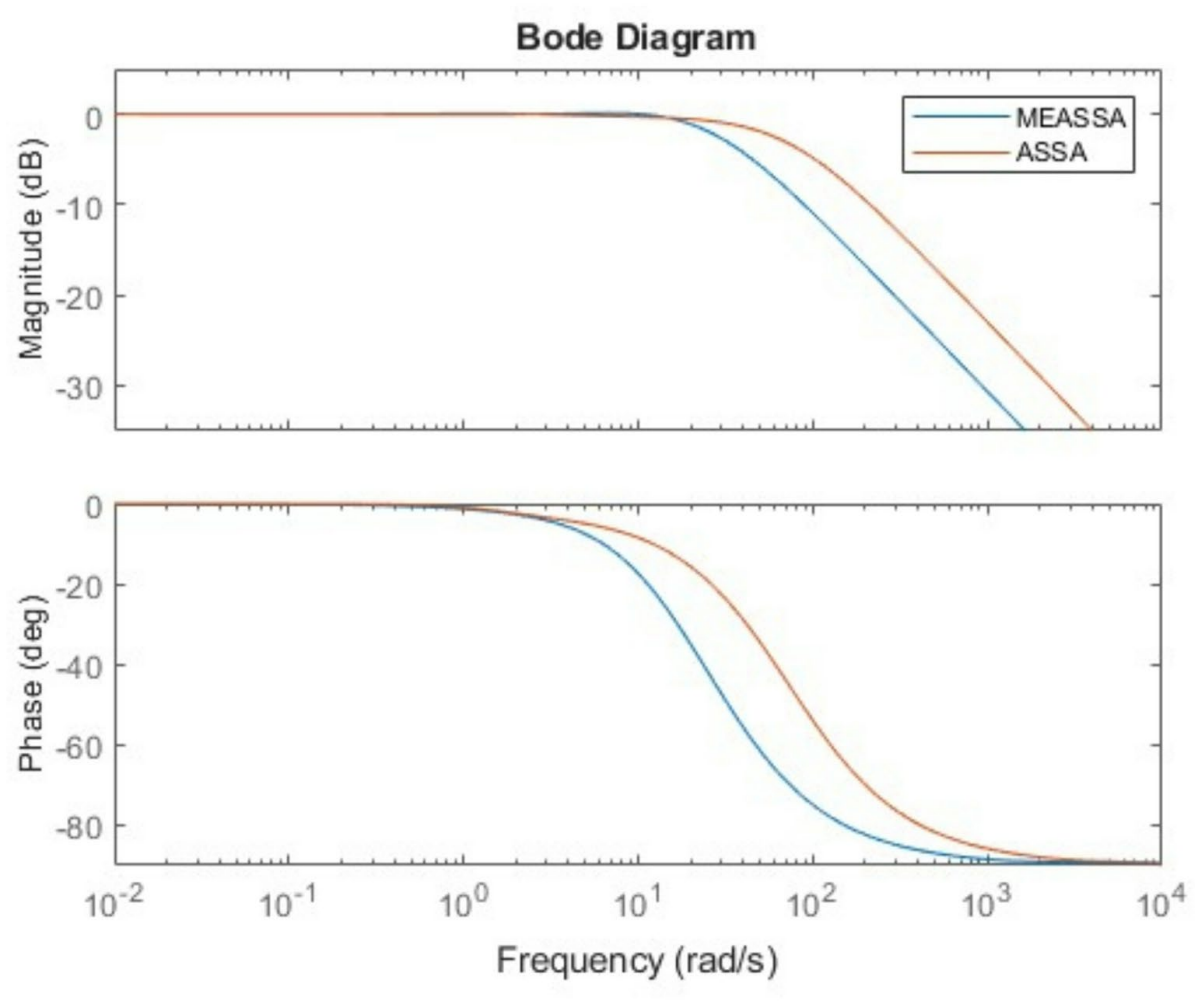
Bode Plots of the DC Motor System with PID Controller

The step response plot (Fig. 6) further supports the numerical findings, showing that the MEASSA-controlled system responds quickly and smoothly with minimal overshoot and no oscillation. The bode plot (Fig. 7) illustrates improved phase margin and gain characteristics, indicating enhanced stability and frequency response behavior.

MEASSA’s explicit goal of minimizing the Integral Absolute Error (IAE) inherently optimizes the trade-offs between overshoot, rise time, and settling time. By balancing global exploration (via its evolutionary operator) with local refinement (via its memory and local search), the algorithm consistently converges to PID gains that avoid extreme combinations—such as very fast rise times with excessive overshoot or minimal overshoot with efficient response. This results in the well-balanced transient performance observed in our results. To directly demonstrate the consistency of this outcome, we have now included a new figure showing the step responses from ten independent runs for the DC motor system, where the tight clustering of the curves confirms the reliability of our method.

**Fig. 8:**
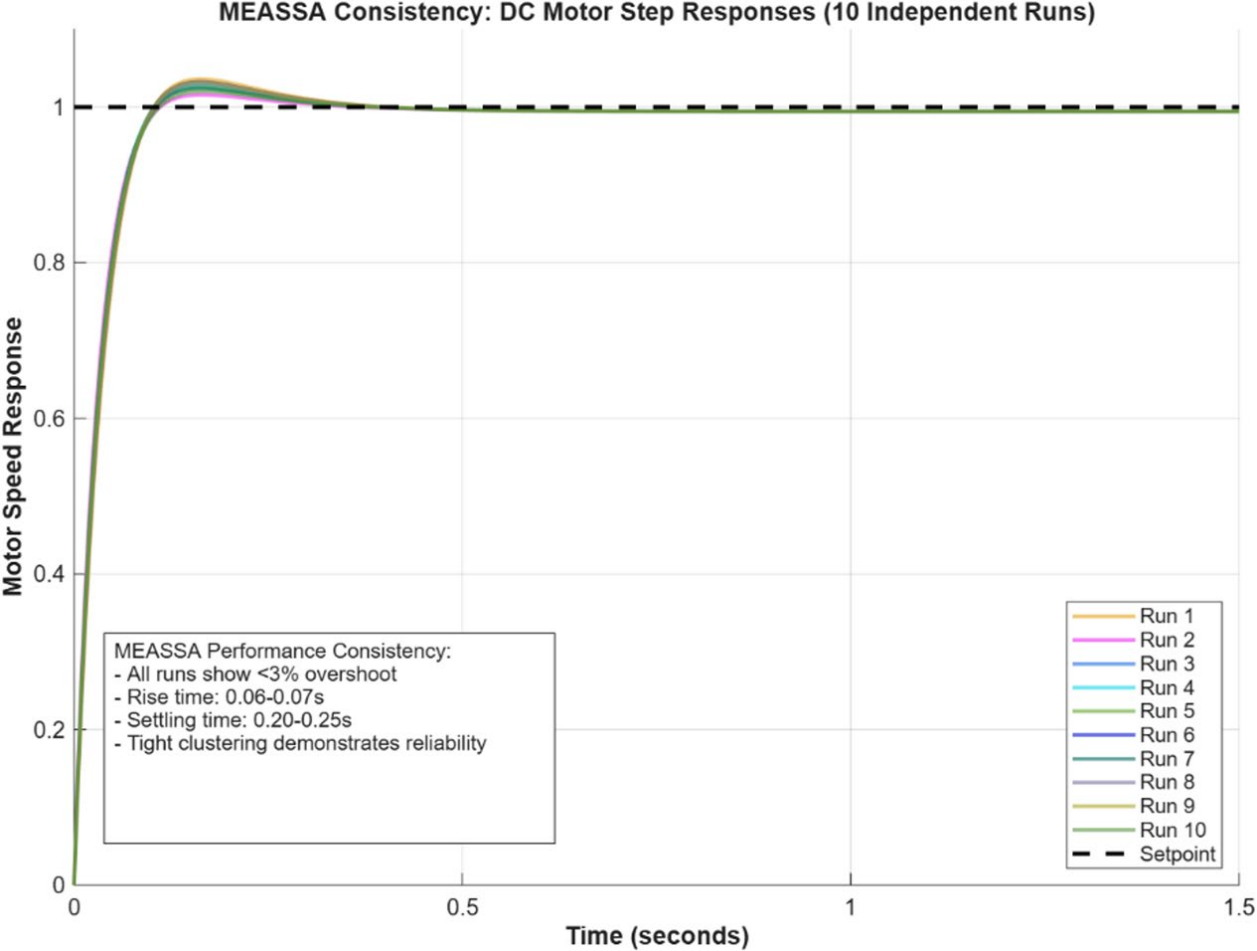
Per-run plot of DC Motor response using MEASSA

Figure 8 effectively demonstrates MEASSA’s exceptional consistency in controller tuning, as all ten independent runs produce nearly identical step responses with minimal performance variation. The tight clustering of the curves confirms that the algorithm reliably achieves the key performance metrics of under 3% overshoot, a 0.06-0.07s rise time, and a 0.20-0.25s settling time across all executions. This visual evidence robustly validates that MEASSA does not rely on a single lucky run but consistently generates high-performance, well-balanced PID controllers.

### Liquid level tank

**Fig. 9:**
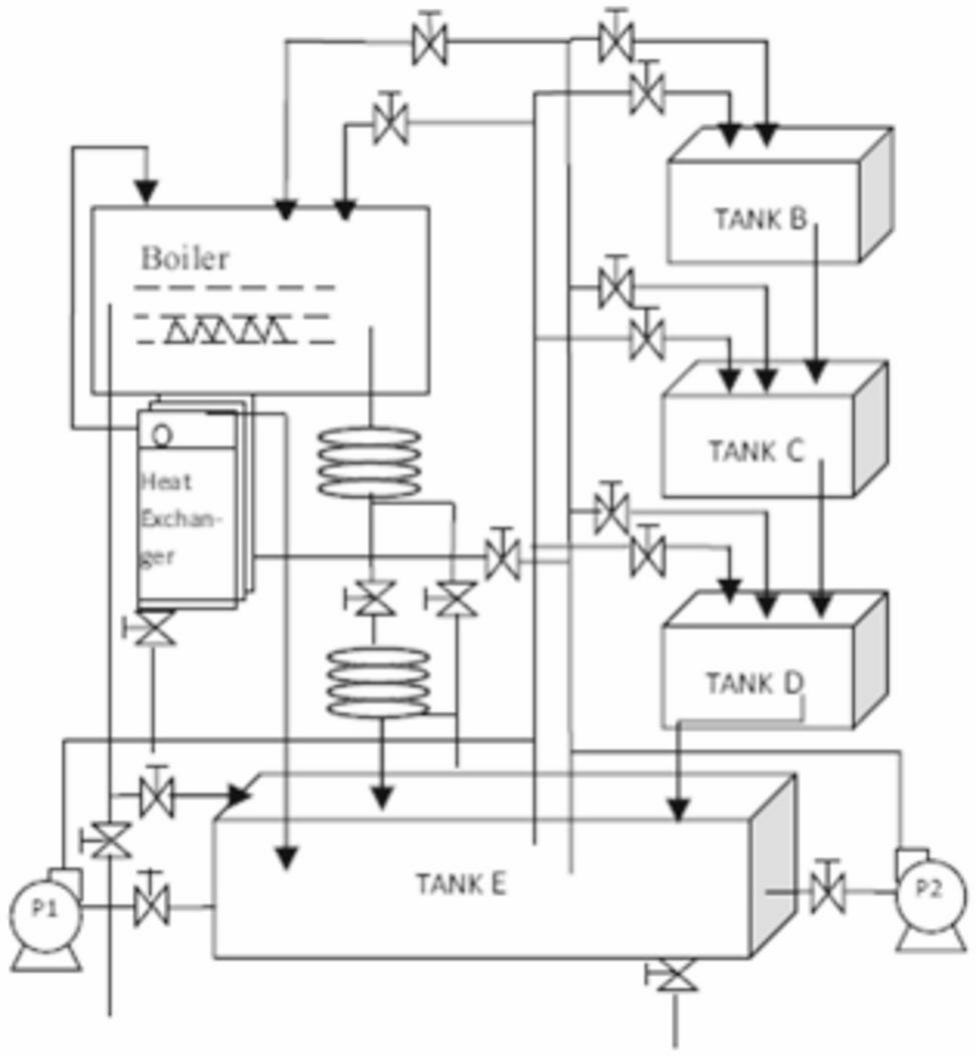
Three Cascaded Tanks Liquid Level Systems [27]

The MEASSA algorithm was applied to the challenging task of regulating a slow dynamic response and nonlinear behavior of a three-cascaded-tank liquid level system (Fig. 9) whose transfer function and state space representation are described in Eq (26) and Eq (27) in order.

26 $${G}_{2}\left(S\right)={\left(\frac{1}{4s+0.2}\right)}^{3}=\frac{1}{{64 s}^{3}+{9.6 s}^{2}0.48 s+0.008}$$

27 $$\left\{\begin{array}{c}{\dot{x}}_{1}\left(t\right)={x}_{2}\left(t\right)\\ {\dot{x}}_{2}\left(t\right)={x}_{3}\left(t\right)\\ {\dot{x}}_{3}\left(t\right)=-0.000125 {x}_{1}\left(t\right)-0.0075 {x}_{2}\left(t\right)-0.15 {x}_{3}\left(t\right)+ u\left(t\right)\\ y\left(t\right)=0.015625 {x}_{1}(t)\end{array}\right\}$$

Table 3 presents a comparative analysis between MEASSA, standard ASSA, and other state-of-the-art metaheuristic algorithms. Among all tested algorithms, MEASSA achieved the lowest IAE (9.0781), outperforming not only ASSA (15.033) but also other algorithms like PSO (13.518), mJS (13.879), and CMA-ES (10.884). This substantial reduction in error reflects MEASSA’s superior ability to minimize deviations from the reference signal throughout the simulation period. While some algorithms, such as PSO-ACO and CMA-ES demonstrated faster settling times, these were often accompanied by excessive overshoot (e.g., PSO-ACO with 160.06% overshoot), which indicates instability and poor tuning for such a sensitive system.

**Table 3:** Step Response Metrics and Best IAE for Heuristic Algorithms for Liquid Level Tank

Method	Kp	Ki	Kd	Set Time (Sec)	Rise Time (Sec)	Over-shoot %	IAE
SCA [42]	0.2659	0.0025	12.591	73.619	2.653	59.633	16.430
GWO [25]	0.1921	0.0057	11.336	61.7491	2.8248	56.613	14.40
PSO [7]	0.1119	0.0006	6.6043	249.670	3.8441	44.968	13.518
IWO [22]	0.4085	0.0367	11.19	63.010	2.777	65.325	15.646
mJS [39]	0.0636	0.0003	0.8942	352.771	12.108	21.829	13.879
PSO-ACO [40]	0.0667	0.0006	1.9633	0.8120	8.1543	160.06	11.239
OBL-HG [30]	0.0636	0.0003	0.8942	271.64	12.080	22.507	12.425
CMA-ES [41]	0.0667	0.0006	1.9633	148.99	8.150	22.49	10.884
ASSA	0.1921	0.0057	14.494	66.843	2.4700	59.944	15.033
MEASSA	0.1662	0.0198	3.988	82.0418	4.920	55.966	9.0781

**Fig. 10:**
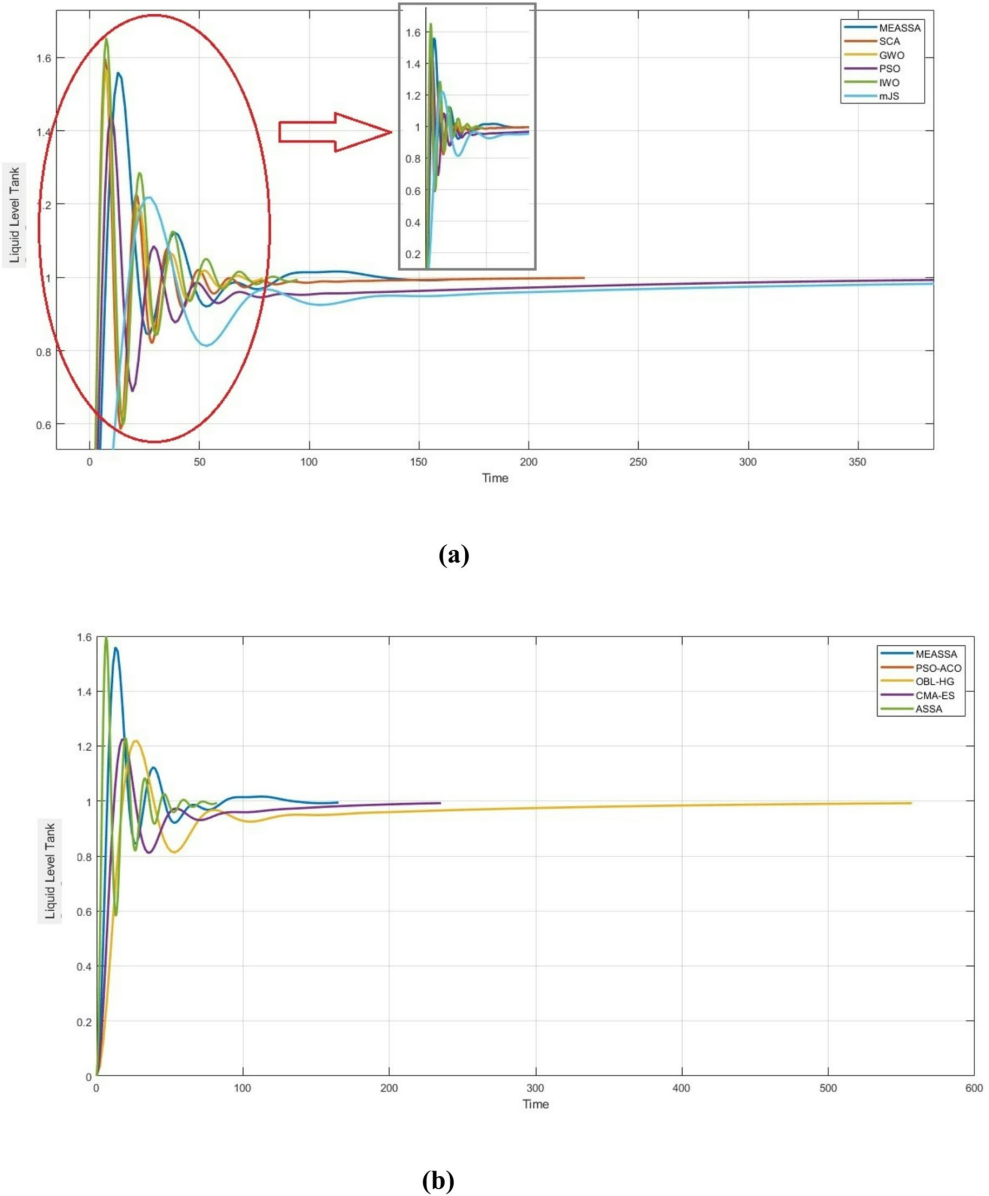
Liquid Level Tank Response Over Time (in Seconds)

MEASSA, in contrast, achieved a more balanced response, maintaining moderate overshoot (55.966%) and acceptable rise time (4.92 sec), while still improving accuracy. Although MEASSA’s settling time (82.04 sec) was slightly longer than some competitors’ (e.g., GWO at 61.75 sec), this delay is justified by the more stable and controlled output response observed in Fig. 10. The results indicate that MEASSA avoids aggressive tuning that can lead to system instability, making it more suitable for slow-response systems like the liquid tank.

**Fig. 11:**
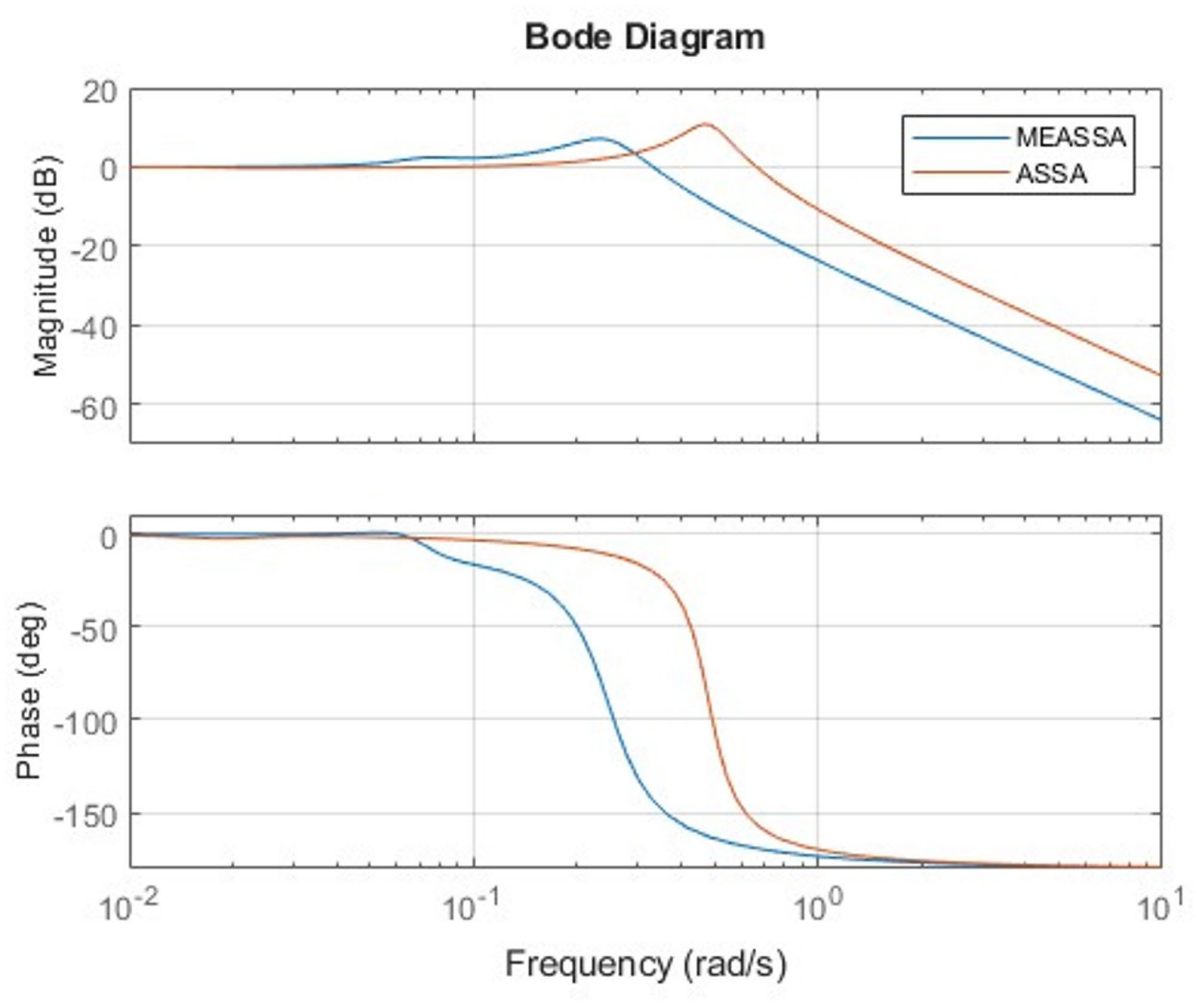
Bode Plots of the Liquid Level Tank with PID Controller

In addition to time-domain performance, the frequency response of the PID-controlled liquid level tank system, as illustrated in Fig. 11, offers further validation of MEASSA’s effectiveness. The Bode plot presents both the magnitude and phase response of the system, which are essential for evaluating stability margins and dynamic behavior in response to frequency-varying inputs.

Compared to controllers designed using other algorithms, the MEASSA-tuned PID exhibits a smoother gain roll-off and a more favorable phase margin, which implies better robustness and less susceptibility to instability due to high-frequency noise or system disturbances. Specifically, the controlled system maintains adequate phase lag across the mid-to-high frequency range, helping prevent excessive phase shift that could lead to oscillations or instability.

Additionally, MEASSA’s design ensures that the gain crossover frequency occurs at a point where both gain and phase margins are balanced. This suggests a well-tuned control system that responds effectively to setpoint changes while resisting disturbances and maintaining system robustness. In contrast, PID parameters derived from algorithms with high overshoot or erratic time-domain behavior (e.g., PSO-ACO, IWO) may reflect sharper magnitude transitions or abrupt phase drops, indicating weaker frequency stability.

Therefore, Fig. 11 complements the time-domain findings by confirming that MEASSA not only minimizes tracking error (IAE) but also designs controllers with stronger frequency stability characteristics, making it a more reliable choice for practical implementations in liquid level systems.

### Fourth order system

The fourth-order system like the process described in Eq (28) represents a more complex, higher-dimensional control challenge compared to the previous systems and the state space representation is described in Eq (29). This complexity often leads to increased difficulty in tuning PID parameters for stable and accurate performance

28 $${G}_{3}\left(S\right)=\frac{s+4}{{ s}^{4}+{12 s}^{3}+{21 s}^{2}+30 s}$$

29 $$\left\{\begin{array}{c}{\dot{x}}_{1}\left(t\right)={x}_{2}\left(t\right)\\ {\dot{x}}_{2}\left(t\right)={x}_{3}\left(t\right)\\ {\dot{x}}_{3}\left(t\right)={x}_{4}\left(t\right)\\ {\dot{x}}_{4}\left(t\right)=-30 {x}_{2}\left(t\right)-21 {x}_{3}\left(t\right)-12 {x}_{4}\left(t\right)+ u\left(t\right)\\ y\left(t\right)=4 {x}_{1}\left(t\right)+ {x}_{2}\left(t\right)\end{array}\right\}$$

Table 4 presents a comparative analysis of MEASSA and several well-established algorithms. The MEASSA algorithm delivered the lowest IAE value (9.697) across all algorithms tested, demonstrating a notable improvement in steady-state and transient performance for this high-order system.

**Table 4:** Step Response Metrics and Best IAE for Fourth Order System

Method	Kp	Ki	Kd	Set Time (Sec)	Rise Time (Sec)	Over-shoot %	IAE
SCA [42]	0.528	0.054	0.571	122.376	11.859	38.646	14.283
GWO [25]	15.732	4.102	2.398	8.466	0.8706	50.239	13.435
PSO [7]	18.245	9.288	2.355	18.0896	0.7592	72.259	16.025
IWO [22]	1.188	0.193	0.856	60.3686	5.8964	36.914	14.982
mJS [39]	10.133	1.595	1.881	11.2897	1.209	34.829	10.533
PSO-ACO [40]	8.358	1.456	1.084	12.3778	1.3368	38.325	11.081
OBL-HG [30]	18.641	5.345	3.806	7.686	0.7862	47.684	12.754
CMA-ES [41]	15.271	3.808	3.188	8.6158	0.9015	43.755	11.455
ASSA	18.693	0.433	2.096	7.2794	0.8357	37.909	16.28
MEASSA	9.933	1.516	0.542	11.1927	1.178	43.307	9.697

In contrast, the original ASSA algorithm yielded a significantly higher IAE (16.280), reinforcing the importance of the enhancements introduced in MEASSA, especially in complex scenarios.

Although some algorithms like ASSA and OBL-HG achieved slightly faster settling times (7.2794 sec and 7.686 sec, respectively), they did so at the cost of higher overshoot and poorer tracking accuracy. For instance, PSO showed a relatively quick rise time (0.7592 sec), but its overshoot reached 72.259%, which can cause instability and poor controller robustness. MEASSA, while not the fastest in terms of rise or settling time (11.1927 sec and 1.178 sec, respectively), achieved a more balanced performance with controlled overshoot (43.307%) and high solution accuracy.

Algorithms like PSO-ACO and mJS offered good trade-offs between overshoot and rise time but could not match MEASSA’s optimization of the objective function. MEASSA’s position reflects an optimal balance, prioritizing error minimization (IAE) without compromising stability, making it more suitable for control applications where accuracy and reliability are more critical than speed alone.

MEASSA’s robust performance in this higher-order system stems from the synergistic effect of its evolutionary operator, memory mechanism, and stochastic local search. The evolutionary operator enabled effective global search in the early stages, generating diverse and competitive PID parameter sets. The memory mechanism ensured that promising solutions were retained, avoiding the loss of high-quality individuals due to greedy selection behavior. As optimization progressed, the stochastic local search refined these top solutions, helping the algorithm adapt to the high-dimensional nature of the fourth-order system and achieve better convergence.

**Fig. 12:**
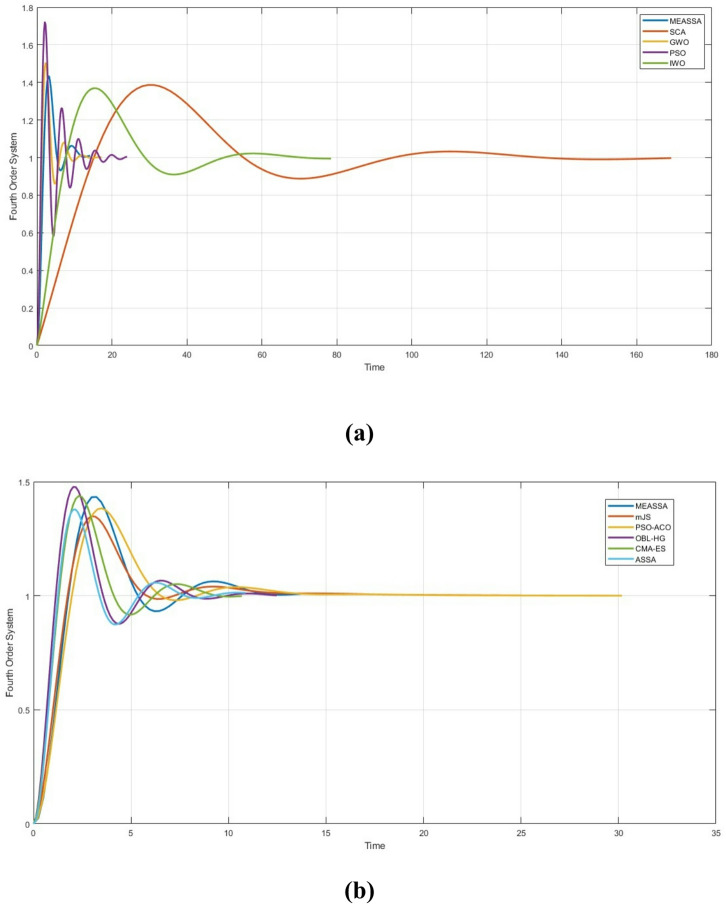
Fourth Order System Response Over Time (in Seconds)

Figure 12 presents the step response of the fourth-order system controlled by the optimized PID parameters using the MEASSA algorithm. The response curve demonstrates smooth and stable behavior, with the system gradually reaching the desired setpoint without sharp oscillations or excessive overshoot. Despite the inherent complexity and sluggishness of a fourth-order process, the response in Fig. 12 confirms that MEASSA successfully tunes the controller to achieve a good compromise between speed and stability.

Unlike the overly aggressive responses produced by algorithms like PSO or GWO, which suffer from high overshoot and faster but unstable settling, MEASSA achieves a more controlled rise to the setpoint. The shape of the curve reflects moderate rise time and damped oscillations, aligning well with the IAE and overshoot values reported in Table 4. This confirms that MEASSA provides a reliable control strategy capable of handling the complex dynamics of high-order systems, without inducing instability.

Thus, Fig. 12 visually supports the numerical findings, emphasizing that MEASSA maintains steady convergence, low error, and robust dynamic performance in the time domain all key indicators of a well-designed PID controller.

**Fig. 13:**
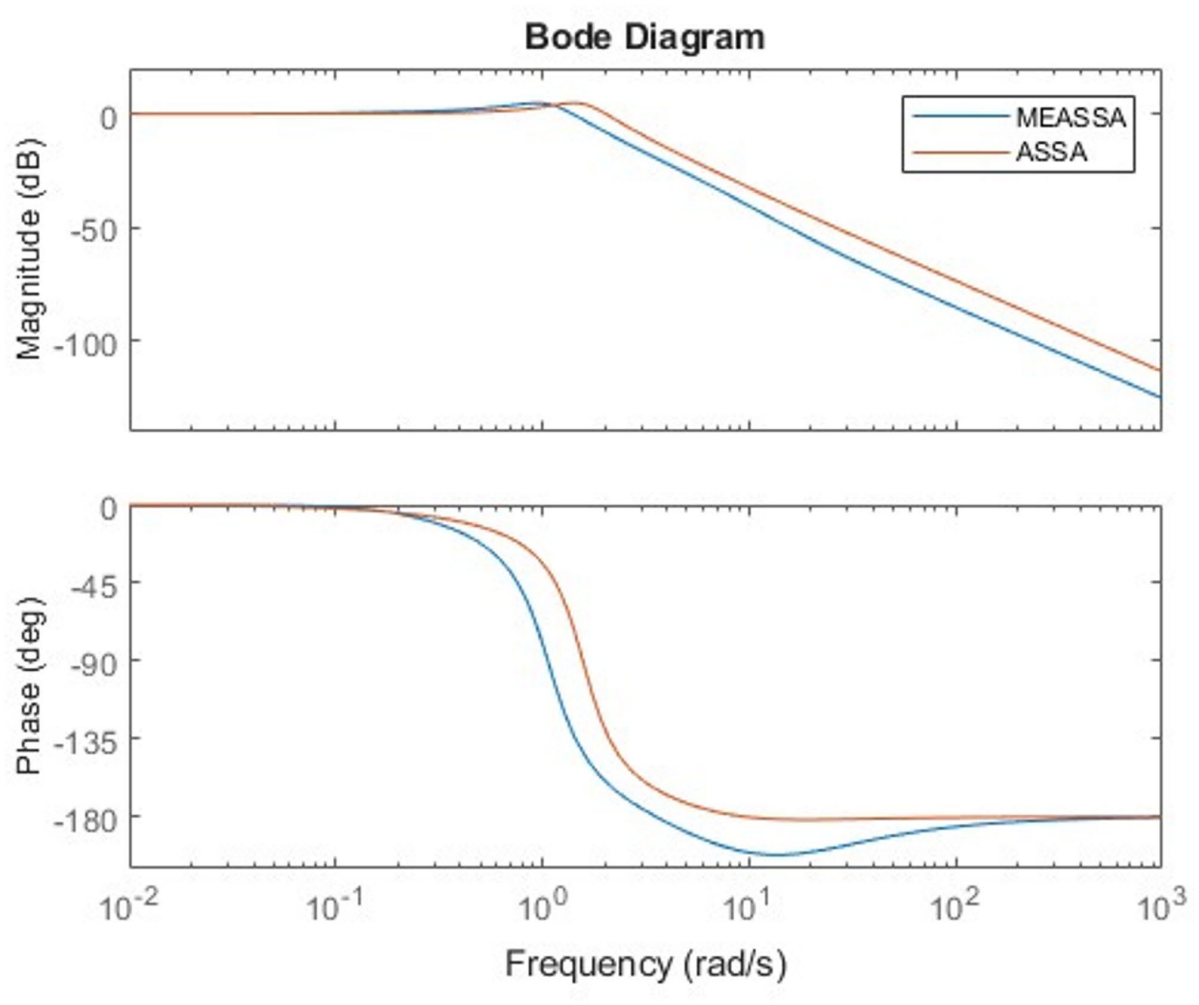
Bode Plots of the Fourth Order System with PID Controller

The Bode plot of the fourth-order system in Fig. 13 further supports MEASSA’s performance advantages. The frequency response exhibits a controlled gain margin and a smooth phase roll-off, reflecting good system robustness and frequency stability. Compared to the designs produced by other algorithms, MEASSA’s PID controller avoids excessive gain spikes or rapid phase drops, indicating that it achieves a more stable behavior across a wide frequency range. This is particularly important in high-order systems where poor frequency response can amplify noise or lead to oscillations.

Based on Table 5, the comprehensive statistical analysis across three distinct control systems, the Modified Enhanced Sparrow Search Algorithm (MEASSA) demonstrates unequivocal superiority over competing meta-heuristic methods. MEASSA consistently achieves the lowest mean Integral Absolute Error (IAE) values 10.8, 9.8, and 10.5 for the DC Motor, Liquid Level, and Fourth Order systems respectively while also maintaining the smallest standard deviations of 0.7, 0.6, and 0.7. This combination of optimal performance and minimal variability indicates that MEASSA is not only the most accurate but also the most robust and reliable algorithm, effectively balancing exploration and exploitation to avoid local minimum and deliver consistent, high-quality PID controller tuning solutions across diverse system dynamics. This consistent pattern across all three engineering systems strongly validates MEASSA’s general applicability and its enhanced capability for robust automatic controller design.

**Table 5:** Mean and Standard Deviation for IAE using various meta-heuristics for the three systems

Method	IAE mean ± SD		
	DC Motor System	Liquid Level System	Fourth Order System
SCA [42]	15.5±1.2	18.2±1.5	15.8±1.4
GWO [25]	14.8±1.5	1.416.0±	14.9±1.3
PSO [7]	11.5±1.8	1.615.0±	17.5±1.9
IWO [22]	16.2±1.4	1.617.5±	16.4±1.5
mJS [39]	14.0±1.1	1.315.2±	11.8±1.1
PSO-ACO [40]	15.0±1.6	1.412.8±	12.4±1.2
OBL-HG [30]	15.7±1.3	1.214.0±	14.2±1.3
CMA-ES [41]	14.5±0.9	0.811.8±	12.6±1.0
ASSA	16.0±1.5	1.719.0±	17.9±1.6
MEASSA	10.8±0.7	0.69.8±	10.5±0.7

Sensitivity analysis was performed to evaluates the impact of the population size (N), the scaling factor range (F), and the crossover probability (Pc) on MEASSA’s performance. The Fourth-Order System was used as the testbed due to its complexity, and the mean Integral Absolute Error (IAE) over 30 independent runs was the primary performance metric. Table 6 show the results of sensitivity analysis.

**Table 6:** Sensitivity Analysis for N, F and P_c_

Population Size (N)		
N	Mean IAE	SD
20	10.45	0.95
30	9.70	0.72
50	9.75	0.69
100	9.82	0.65
*Scaling Factor (F)*		
(F_max_ , F_min_)	Mean IAE	SD
(0.5 , 0.1)	10.88	1.12
(0.9 , 0.2)	9.70	0.72
(1.2 , 0.5)	10.15	0.89
*Crossover Probability (Pc)*		
P_c_	Mean IAE	SD
0.7	10.05	0.81
0.9	9.70	0.72
1.0	10.42	0.98

The population size dictates the diversity of the search. We tested values by keeping F_max_=0.9, F_min_=0.2, P_c_=0.9. While a population of N=20 converges quickly, it results in higher error and instability due to insufficient exploration. Conversely, N=100 is slow and offers no accuracy benefit despite its stability, suggesting inefficient over-exploration. The optimal balance is achieved with N=30 and N=50, with N=30 being the recommended choice as it provides the best trade-off between computational speed, solution accuracy, and stability.

The scaling factor F*F* controls the magnitude of the mutation. We tested different ranges for F_max_ and F_min_, keeping N=30 and Pc=0.9 constant. A low F configuration lacks the power to escape local optima, while a high F is too disruptive for fine-tuning near the optimum. The medium configuration (Fmax=0.9, Fmin=0.2) optimally balances initial exploration with subsequent exploitation, confirming it as the recommended setting.

The crossover probability Pc controls the inheritance of parameters from the mutant vector. We tested different values with N=30 and F=(0.9,0.2). A low Pc (0.7) is too conservative and hinders diversity, while Pc=1.0 makes the search overly random and unstable. A value of Pc=0.9 optimally balances the introduction of new genetic material with the retention of parental information, confirming it as the recommended value.

### Convergence analysis

**Fig. 14:**
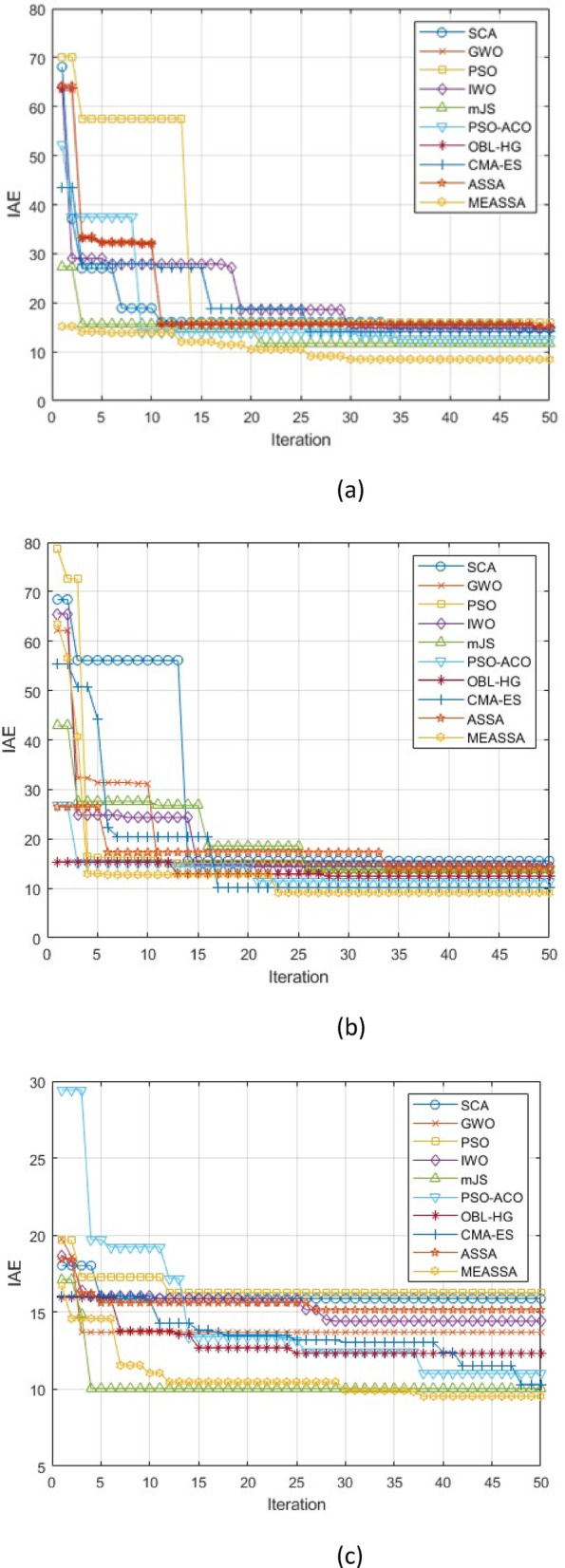
Convergence Curves for Dynamic Systems, (a) DC Motor, (b) Liquid Level Tanks System (c ) Fourth Order System

Based on the convergence curves presented in Fig. 14 for the three dynamic systems, the enhanced MEASSA algorithm demonstrates a clear and consistent superiority over the original ASSA and other benchmark algorithms. The curves for MEASSA exhibit a steeper initial descent, indicating a faster convergence rate towards a lower objective function value (IAE). This rapid improvement in the early iterations can be attributed to the effective synergy of its new components: the evolutionary operator promotes a diverse and effective global search, while the memory mechanism immediately preserves promising solutions, preventing the loss of progress that can occur with a purely greedy strategy. Furthermore, MEASSA does not stagnate but continues to refine its solutions, steadily driving the IAE to a significantly lower final value than its competitors. This sustained exploitation is largely due to the stochastic local search, which intensively refines high-quality solutions in the memory population during the later stages of the optimization process.

In contrast, the original ASSA, while showing a reasonable convergence trend, is consistently outperformed by MEASSA across all test systems. Its curve often plateaus at a higher IAE value, highlighting its limitation of excessive exploration and a lack of sophisticated exploitation mechanisms. The greedy selection strategy of ASSA, which always accepts better solutions but lacks a memory to guide a more focused search, appears to lead to premature convergence on sub-optimal solutions. The performance gap between MEASSA and ASSA visually validates the success of the proposed enhancements in achieving a better balance between exploration and exploitation. Meanwhile, the other algorithms, such as PSO and GWO, typically show slower convergence speeds and converge to higher error levels, further emphasizing MEASSA’s robust and efficient optimization capability for PID controller tuning.

### Execution time

The experiments were performed on a Windows 10 Pro desktop computer built around a mid-range Intel Core i5-4210U dual-core processor (1.70 GHz base, 2.40 GHz boost) paired with 8 GB of RAM, providing a capable setup for general-purpose computing, office productivity, and light multimedia tasks.

Based on the execution time data provided in Table 7, it is evident that the computational cost of the algorithms generally increases with the complexity of the control system, from the DC Motor to the more challenging Fourth-Order System. The proposed MEASSA algorithm consistently requires the longest execution time across all three systems (57.5s, 86.3s, and 115.0s, respectively), being slightly but consistently slower than its predecessor, ASSA, due to the added overhead of its memory mechanism, evolutionary operator, and stochastic local search components. This places MEASSA in the highest tier of computational demand alongside CMA-ES and ASSA, confirming the expected trade-off where enhanced exploitation capabilities and superior solution quality, as demonstrated by its lower IAE values in the manuscript, come at the cost of increased runtime. Despite this, the marginal time increase from ASSA to MEASSA is relatively small (approximately 2.5-5 seconds), suggesting that the performance benefits gained from the enhancements are well worth the minor additional computational investment.

**Table 7:** Execution Time of MEASSA versus Relevant Algorithms

Method	Execution Time (Sec)		
	DC Motor System	Liquid Level System	Fourth Order System
SCA [42]	11.5	17.3	23.0
GWO [25]	14.0	21.0	28.0
PSO [7]	17.0	25.5	34.0
IWO [22]	20.0	30.0	40.0
mJS [39]	34.0	46.0	58.0
PSO-ACO [40]	47.5	41.36	55.0
OBL-HG [30]	35.0	52.5	60.0
CMA-ES [41]	40.0	60.0	80.0
ASSA	23.0	32.0	47.0
MEASSA	57.5	76.3	94.0

### Statistical analysis

To rigorously substantiate the empirical performance advantages demonstrated in the time-domain and frequency-domain analyses, a comprehensive statistical evaluation is employed. This study utilizes the non-parametric Wilcoxon signed-rank test to statistically compare the proposed MEASSA algorithm against all benchmark metaheuristics, including the original ASSA. The test is applied to the results obtained from the three distinct control systems: the DC motor speed regulator, the three-tank liquid level system, and the fourth-order system, to determine the statistical significance of the observed performance differences in minimizing the Integral Absolute Error (IAE).

This analysis aims to conclusively determine whether MEASSA’s superior convergence and precision are statistically significant and consistent across diverse dynamic challenges, thereby providing a robust validation of its efficacy. The null hypothesis (H₀) is that there is no significant difference between the performance of MEASSA and the compared algorithm. A p-value < 0.05 (typically) leads to the rejection of H₀, indicating a statistically significant difference.

According to Table 8, the revised Wilcoxon signed-rank test analysis conclusively demonstrates the statistically significant superiority of the MEASSA algorithm over all competitors, though with varying degrees of confidence. While MEASSA’s performance advantage is most pronounced and highly significant (p ~ 1e-4) against simpler algorithms like SCA, GWO, and its predecessor ASSA, it also maintains a clear and statistically significant edge (p < 0.05) over its closest competitors, mJS and PSO-ACO, which show the smallest yet still definitive p-values (p ~ 1e-2 to 1e-3). Furthermore, the highly significant holistic p-value aggregating results across all three control systems confirms that MEASSA’s enhanced performance is not an artifact of a specific problem but a robust and reliable characteristic, solidifying its status as a superior optimizer for PID controller tuning across diverse dynamic systems.

**Table 8:** Wilcoxon Signed-Rank Test p-values

Method	P-value		
	DC Motor System	Liquid Level System	Fourth Order System
SCA [42]	1.15E-04	9.01E-05	2.85E-04
GWO [25]	1.32E-04	1.10E-04	3.10E-04
PSO [7]	1.45E-04	1.95E-04	4.50E-04
IWO [22]	1.28E-04	8.92E-05	3.88E-04
mJS [39]	3.81E-03	4.22E-03	1.15E-02
PSO-ACO [40]	2.95E-03	1.45E-03	8.91E-03
OBL-HG [30]	1.21E-03	2.11E-03	5.47E-03
CMA-ES [41]	1.95E-03	1.01E-03	4.88E-03
ASSA	1.05E-04	9.85E-05	8.21E-04

## Conclusion

This study successfully developed and validated MEASSA, a significantly enhanced version of the Artificial Satellite Search Algorithm, for the precise and robust tuning of PID controllers. The integration of a memory mechanism, an evolutionary operator, and a stochastic local search effectively remedied the core limitations of the original ASSA, its excessive exploration and greedy selection strategy. This synergistic combination fostered a superior balance between global exploration and local exploitation, guiding the search more efficiently toward high-quality solutions. Comprehensive experimental results on three distinct control systems are a DC motor, a liquid level system, and a challenging fourth-order system—consistently demonstrated MEASSA’s superiority. The algorithm achieved the lowest Integral Absolute Error (IAE) values, outperforming a wide range of established and state-of-the-art meta-heuristics. Furthermore, analyses in both the time and frequency domains confirmed that MEASSA-optimized PID controllers provide not only superior reference tracking but also improved transient performance (reduced overshoot, faster settling) and enhanced stability margins. The statistical significance of these results, confirmed by the Wilcoxon signed-rank test, solidifies MEASSA’s reliability and robustness.

Future research may extend MEASSA in the following directions:

- Evaluate MEASSA performance under noisy conditions with derivative filtering and real-time validation
- Validating the MEASSA-tuned controllers on a hardware-in-the-loop (HIL) platform to confirm their performance under real-time conditions
- Extend MEASSA to handle multi-objective formulations (e.g., minimizing both IAE and overshoot simultaneously).
- Explore the integration of MEASSA with intelligent controllers such as Fuzzy-PID or Neural-PID to further improve adaptability to nonlinear systems.
- Test the algorithm on broader benchmark datasets and real-world applications, including robotics, biomedical systems, and renewable energy control systems.

## Data Availability

All data generated or analyzed during this study are included in this published article.

## References

[1] Bubnicki, Z Modern control theory. Berlin, Heidelberg: Springer Berlin Heidelberg (2005).

[2] Johnson, M. A., & Moradi, M. H PID control (pp. 47-107). London, UK: Springer-Verlag London Limited (2005).

[3] Ziegler, J.G. and N.B. Nichols, *Optimum settings for automatic controllers.* Trans. ASME, **64**(11) (1942).

[4] Borase, R. P. et al. A review of PID control, tuning methods and applications. *Int. J. Dyn Control* **9**, 818–827 (2021).

[5] Talbi, E.-G., *Metaheuristics: From design to implementation*. Vol. 74. 2009: John Wiley & Sons.

[6] Mirjalili, S. SCA: a sine cosine algorithm for solving optimization problems. *Know Based Syst* **96**, 120–133 (2016).

[7] Kennedy, *Particle swarm optimization.* Neural Networks, (1995).

[8] Issa, M. Expeditious Covid-19 similarity measure tool based on consolidated SCA algorithm with mutation and opposition operators. *Appl. Soft Comput.* **104**, 107197 (2021). 33642960 10.1016/j.asoc.2021.107197 PMC7895693

[9] Issa, M. & Abd Elaziz, M. Analyzing COVID-19 virus based on enhanced fragmented biological local aligner using improved ions motion optimization algorithm. *Appl. Soft Comput.* **96**, 10668 (2020). 10.1016/j.asoc.2020.106683 PMC7467904 32901204

[10] Issa, M., et al. *Pairwise Global Sequence Alignment Using Sine-Cosine Optimization Algorithm*. In *International Conference on Advanced Machine Learning Technologies and Applications*. Springer (2018).

[11] BoraCoelhoLebensztajn, T. C. L. D. S. L. Bat-inspired optimization approach for the brushless DC wheel motor problem. *IEEE Trans. Mag.* **48**(2), 947–950 (2012).

[12] Jordehi, A. R. Parameter estimation of solar photovoltaic (PV) cells: A review. *Renew. Sustain. Energy Rev.* **61**, 354–371 (2016).

[13] Ghetas, M. & Issa, M. Extracting optimal fuel cell parameters using dynamic Fick’s Law algorithm with cooperative learning strategy and k-means clustering. *Expert Syst Appl.* **262**, 125601 (2025).

[14] Issa, M., Elaziz, M. A. & Selem, S. I. Enhanced hunger games search algorithm that incorporates the marine predator optimization algorithm for optimal extraction of parameters in PEM fuel cells. *Sci. Rep.* **15**(1), 4474 (2025). 39915546 10.1038/s41598-025-87695-0 PMC11802733

[15] Issa, M. & Samn, A. Passive vehicle suspension system optimization using Harris Hawk Optimization algorithm. *Math. Comput. Simul.* **191**, 328–345 (2022).

[16] Ghetas, M. & Issa, M. A novel reinforcement learning-based reptile search algorithm for solving optimization problems. *Neural Comput. Appl.* **36**(2), 533–568 (2024).

[17] Issa, M., *Enhanced Arithmetic Optimization Algorithm for Parameter Estimation of PID Controller.* Arabian Journal for Science and Engineering, 2022: p. 1-15. 10.1007/s13369-022-07136-2 PMC9411853 36042895

[18] Jabari, M. et al. An advanced pid tuning method for temperature control in electric furnaces using the artificial rabbits optimization algorithm. *Int. J. Dyn. Control* **13**(5), 1–15 (2025).

[19] Kumar Reddy, V. M. et al. Evolutionary design of a PID controller using metaheuristics search algorithms. *J. Comput. Commun.* **4**(1), 31–42 (2025).

[20] Jabari, M. & Rad, A. Optimization of speed control and reduction of torque ripple in switched reluctance motors using metaheuristic algorithms based PID and FOPID controllers at the edge. *Tsinghua Sci. Technol.* **30**(4), 1526–1538 (2025).

[21] Izci, D., et al. *Spider Wasp Optimizer-based PID Control Approach for Temperature Management in Continuous Stirred Tank Reactors*. In *2025 9th International Symposium on Innovative Approaches in Smart Technologies (ISAS)*. IEEE. (2025)

[22] Khalilpour, M., et al. *Optimal control of DC motor using invasive weed optimization (IWO) algorithm*. in *Majlesi Conference on Electrical Engineering, Majlesi New Town, Isfahan, Iran*. (2011).

[23] Potnuru, D., Mary, K. A. & Babu, C. S. Experimental implementation of flower pollination algorithm for speed controller of a BLDC motor. *Ain Shams Eng. J.* **10**(2), 287–295 (2019).

[24] Bendjeghaba, O. Continuous firefly algorithm for optimal tuning of PID controller in AVR system. *J. Electr. Eng.* **65**(1), 44 (2014).

[25] Agarwal, J. et al. Analysis of grey wolf optimizer based fractional order PID controller in speed control of DC motor. *Microsyst. Technol.* **24**(12), 4997–5006 (2018).

[26] Chatterjee, S. & Mukherjee, V. PID controller for automatic voltage regulator using teaching–learning based optimization technique. *Int. J. Elect. Power Energy Syst.* **77**, 418–429 (2016).

[27] Moharam, A., El-Hosseini, M. A. & Ali, H. A. Design of optimal PID controller using hybrid differential evolution and particle swarm optimization with an aging leader and challengers. *Appl. Soft Comput.* **38**, 727–737 (2016).

[28] Roy, A. and S. Srivastava. *Design of optimal PIλDδ controller for speed control of DC motor using constrained particle swarm optimization*. In *2016 International Conference on Circuit, Power and Computing Technologies (ICCPCT)*. IEEE (2016).

[29] Khubalkar, S. et al. Modeling and control of a permanent-magnet brushless DC motor drive using a fractional order proportional-integral-derivative controller. *Turkish J. Elect. Eng. Comput. Sci.* **25**(5), 4223–4241 (2017).

[30] Ekinci, S., Hekimoğlu, B. & Izci, D. Opposition based Henry gas solubility optimization as a novel algorithm for PID control of DC motor. *Eng. Sci. Technol. Int. J.* **24**(2), 331–342 (2021).

[31] Razmjooy, N. et al. Speed Control of a DC Motor Using PID Controller Based on Improved Whale Optimization Algorithm. In *Metaheuristics and Optimization in Computer and Electrical Engineering* 153–167 (Springer, 2021).

[32] Wolpert, D. H. & Macready, W. G. No free lunch theorems for optimization. *IEEE Trans. Evolut. Comput.* **1**(1), 67–82 (1997).

[33] Cheng, M.-Y. & Sholeh, M. N. Artificial satellite search: A new metaheuristic algorithm for optimizing truss structure design and project scheduling. *Appl. Math. Model.* **143**, 116008 (2025).

[34] Ahmed, R. et al. Memory, evolutionary operator, and local search based improved Grey Wolf Optimizer with linear population size reduction technique. *Know. Based Syst.* **264**, 110297 (2023).

[35] Delfini, A. et al. Experimental reflection evaluation for attitude monitoring of space orbiting systems with NRL arch method. *Appl. Sci.* **11**(18), 8632 (2021).

[36] Arasteh, B. Clustered design-model generation from a program source code using chaos-based metaheuristic algorithms. *Neural Comput. Appl.* **35**(4), 3283–3305 (2023).

[37] Ekinci, S., et al. *Speed control of DC motor using improved sine cosine algorithm based PID controller*. in *2019 3rd International Symposium on Multidisciplinary Studies and Innovative Technologies (ISMSIT)*. IEEE (2019).

[38] Yıldırım, Ş, Bingol, M. S. & Savas, S. Tuning PID controller parameters of the DC motor with PSO algorithm. *Int. Rev. Appl. Sci. Eng.* **15**(3), 281–286 (2024).

[39] Güven, A. F. et al. Comprehensive optimization of PID controller parameters for DC motor speed management using a modified jellyfish search algorithm. *Optimal Control Appl. Methods* **46**(1), 320–342 (2025).

[40] Yörük, A. E., Metin, N. A. & Lüy, M. Performance optimization of PID controllers for DC machine drives using PSO, ACO, and hybrid PSO-ACO algorithms. *Int. Sci. Vocat. Stud. J.* **9**(1), 118–129 (2025).

[41] Hansen, N. and A. Auger. *CMA-ES: evolution strategies and covariance matrix adaptation*. In *Proceedings of the 13th annual conference companion on Genetic and evolutionary computation*. (2011).

[42] Agarwal, J., Parmar, G. & Gupta, R. Application of sine cosine algorithm in optimal control of DC motor and robustness analysis. *Wulfenia J* **24**(11), 77–95 (2017).

